# T‐Cell Exhaustion in the Tumor Microenvironment: Subcellular Dysfunction, Pan‐Cancer Characteristics, and Therapeutic Interventions

**DOI:** 10.1002/advs.75651

**Published:** 2026-05-14

**Authors:** Mingxing Wang, Wanhui Dong, Jian Chen, Zhangjie Zhou, Shujuan Fu, Tingting Wu, Haiyan Jiang, Zhiying Wang, Zhixian Zhong, Yi Zhong

**Affiliations:** ^1^ Shanghai TCM‐integrated Hospital Shanghai University of TCM Shanghai China; ^2^ Shanghai University of Traditional Chinese Medicine Shanghai China; ^3^ Lu'an Hospital of Traditional Chinese Medicine Affiliated to Anhui University of Chinese Medicine Lu'an Anhui China; ^4^ Tongji University Shanghai China

**Keywords:** engineering strategies, immunotherapy, nanomedicine, organelle crosstalk, subcellular remodeling, T‐cell exhaustion

## Abstract

Although immune checkpoint blockade (ICB) therapy has profoundly reshaped the landscape of oncology, T‐cell exhaustion (Tex) remains a core challenge in immunotherapy for solid tumors. Current research predominantly focuses on signal transduction and epigenetic regulation, whereas the adaptive alterations and dysfunction of subcellular organelles within T cells under the stress of the tumor microenvironment (TME) have not been systematically elucidated. This review proposes that suborganellar dysfunction serves as a key functional link in the progression of Tex. We systematically explore the role of dysregulated organelle interaction networks in this exhaustion, including mitochondrial dynamics and metabolic perturbations, aberrant endoplasmic reticulum (ER) stress responses, and mechanical stress‐induced nuclear damage, elucidating how these alterations form a self‐sustaining vicious cycle. Furthermore, we summarize the heterogeneity and commonalities of T‐cell subcellular dysfunction across various solid tumors, such as oxidative stress‐mediated mitochondrial damage in lung cancer, aberrant lipid metabolism‐induced ER stress in hepatocellular carcinoma, and the suppression of lysosomal function in highly glycolytic tumors. Finally, we review emerging interventional strategies targeting these organelle checkpoints, such as nanomaterial‐based mitochondrial protection, delivery systems modulating ER homeostasis, and stimuli‐responsive matrix regulation technologies, aiming to provide novel perspectives for enhancing the anti‐tumor efficacy of T cells via subcellular engineering approaches.

## Graphical abstract

This study elucidates the mechanisms of subcellular multidimensional collapse in exhausted T cells. By specifically targeting the nucleus, mitochondria, and endoplasmic reticulum, strategic interventions can effectively remodel the compromised organelle network. This integrated approach drives comprehensive T cell resuscitation, ultimately establishing robust and durable antitumor immunity.

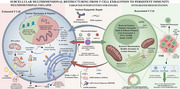

**Abbreviations**
Δψm: mitochondrial membrane potential
ACAT1: acyl‐CoA:cholesterol acyltransferase 1
AMPK: MP‐activated protein kinase
ANT: adenine nucleotide translocator
AP‐1: activator protein‐1
APCs: antigen‐presenting cells
ATF4: activating transcription factor 4
ATF6: activating transcription factor 6
BiP: binding immunoglobulin protein
Blimp‐1: B lymphocyte‐induced maturation protein‐1
BMM: bone marrow microenvironment
Ca^2^^+^
: calcium ion
CAFs: cancer‐associated fibroblasts
CAR: chimeric antigen receptor
ccRCC: clear cell renal cell carcinoma
cGAS: cyclic GMP‐AMP synthase
CHOP: C/EBP homologous protein
CLL: chronic lymphocytic leukemia
c‐Myc: cellular myelocytomatosis oncogene
CRC: colorectal cancer
CRT: calreticulin
CTLA‐4: cytotoxic T‐lymphocyte‐associated protein 4
CTLs: cytotoxic T lymphocytes
DAMPs: damage‐associated molecular patterns
dCas9: catalytically dead Cas9
DCs: dendritic cells
DnaJC15: DnaJ heat shock protein family member C15
Dnmt3a: DNA methyltransferase 3 alpha
Drp1: dynamin‐related protein 1
DSBs: double‐strand breaks
ECM: extracellular matrix
eIF2α: eukaryotic initiation factor 2 alpha
Eomes: eomesodermin
ER: endoplasmic reticulum
ERK1/2: extracellular signal‐regulated kinase 1/2
ETC: electron transport chain
FAM134B: family with sequence similarity 134 member B
FAO: fatty acid oxidation
FATP2: fatty acid transport protein 2
Flcn: folliculin
FoxP3: forkhead box P3
GAP: GTPase‐activating protein
GCN2: general control nonderepressible 2
GLS: glutaminase
Glut1: glucose transporter 1
GLUTs: glucose transporters
GPX4: glutathione peroxidase 4
GRP78: glucose‐regulated protein 78
GSH: glutathione
GZMB: granzyme B gene
HCC: hepatocellular carcinoma
HDACs: histone deacetylases
Helios: Ikaros family transcription factor Helios
HIF‐1α: hypoxia‐inducible factor 1‐alpha
HMGB1: high mobility group box 1
ICB: immune checkpoint blockade
ICD: immunogenic cell death
ICIs: immune checkpoint inhibitors
IFN: interferon
IFNG: interferon‐gamma gene
IFP: interstitial fluid pressure
IL‐10: interleukin‐10
IL‐2: interleukin‐2
IMM: inner mitochondrial membrane
IP3R: inositol 1,4,5‐trisphosphate receptor
IRE1α: inositol‐requiring enzyme 1 alpha
IS: immune synapse
ISR: integrated stress response
KIRA6: kinase inhibiting RNase attenuator 6
LADs: lamina‐associated domains
LAG‐3: lymphocyte‐activation gene 3
LDH: lactate dehydrogenase
LINC complex: Linker of Nucleoskeleton and Cytoskeleton complex
LOX: lysyl oxidase
MAMs: mitochondria‐associated membranes
MCJ: methylation‐controlled J protein
MCT1: monocarboxylate transporter 1
MCTs: monocarboxylate transporters
Mdivi‐1: mitochondrial division inhibitor 1
MDSCs: myeloid‐derived suppressor cells
Mfn2: mitofusin‐2
MHC II: major histocompatibility complex class II
MM: multiple myeloma
MMP: mitochondrial membrane potential
mPTP: mitochondrial permeability transition pore
MSS: microsatellite stable
mtDNA: mitochondrial DNA
MTOC: microtubule‐organizing center
mTORC1: mechanistic target of rapamycin complex 1
NAD^+^
: nicotinamide adenine dinucleotide
NADH: reduced nicotinamide adenine dinucleotide
NFAT: nuclear factor of activated T cells
NR4A: nuclear receptor subfamily 4 group A
NSCLC: non‐small cell lung cancer
OMA1: mitochondrial metalloprotease
Opa1: optic atrophy 1
ORP: oxysterol‐binding protein‐related protein
ORR: objective response rate
OVs: oncolytic viruses
oxLDL: oxidized low‐density lipoprotein
OXPHOS: oxidative phosphorylation
PCa: prostate cancer
PD‐1: programmed cell death protein 1
PDAC: pancreatic ductal adenocarcinoma
PDCD1: programmed cell death protein 1 gene
PDI: protein disulfide isomerase
PD‐L1: programmed death‐ligand 1
PDPA: poly(2‐(diisopropylamino)ethyl methacrylate)
PERK: protein kinase R‐like ER kinase
PFK: phosphofructokinase
PGC1α: peroxisome proliferator‐activated receptor gamma coactivator 1‐alpha
PGE_2_
: prostaglandin E2
PHD: prolyl hydroxylase
PINK1: PTEN‐induced kinase 1
PROTAC: proteolysis‐targeting chimera
RIDD: regulated IRE1‐dependent decay
ROS: reactive oxygen species
SATB1: special AT‐rich sequence‐binding protein 1
SCFAs: short‐chain fatty acids
SERCA: sarco/endoplasmic reticulum Ca^2^^+^‐ATPase
SFAs: saturated fatty acids
Sirtuins: silent information regulators
SLC38A9: solute carrier family 38 member 9
SRC: spare respiratory capacity
STING: stimulator of interferon genes
TCR: T‐cell receptor
Terminal Tex: terminally exhausted subset
Tex: T‐cell exhaustion
TFEB: transcription factor EB
TGF‐β: transforming growth factor‐beta
Th1: T helper 1
TILs: tumor‐infiltrating lymphocytes
TIM‐3: T‐cell immunoglobulin and mucin‐domain containing‐3
TKIs: tyrosine kinase inhibitors
TMB: tumor mutational burden
TME: tumor microenvironment
Tmem: memory T cells
TNBC: triple‐negative breast cancer
TNF‐α: tumor necrosis factor‐alpha
TOX: thymocyte selection‐associated high mobility group box
Tpex: progenitor exhausted subset
Tregs: regulatory T cells
TREX1: three‐prime repair exonuclease 1
TRM: tissue‐resident memory T cells
Trm: tissue‐resident memory T cells
TUDCA: tauroursodeoxycholic acid
UPR: unfolded protein response
VAP: vesicle‐associated membrane protein‐associated protein
V‐ATPase: vacuolar H^+^‐ATPase
VDAC: voltage‐dependent anion channel
XBP1: X‐box binding protein 1
XBP1s: spliced X‐box binding protein 1

## 1 Introduction

**FIGURE 1:**
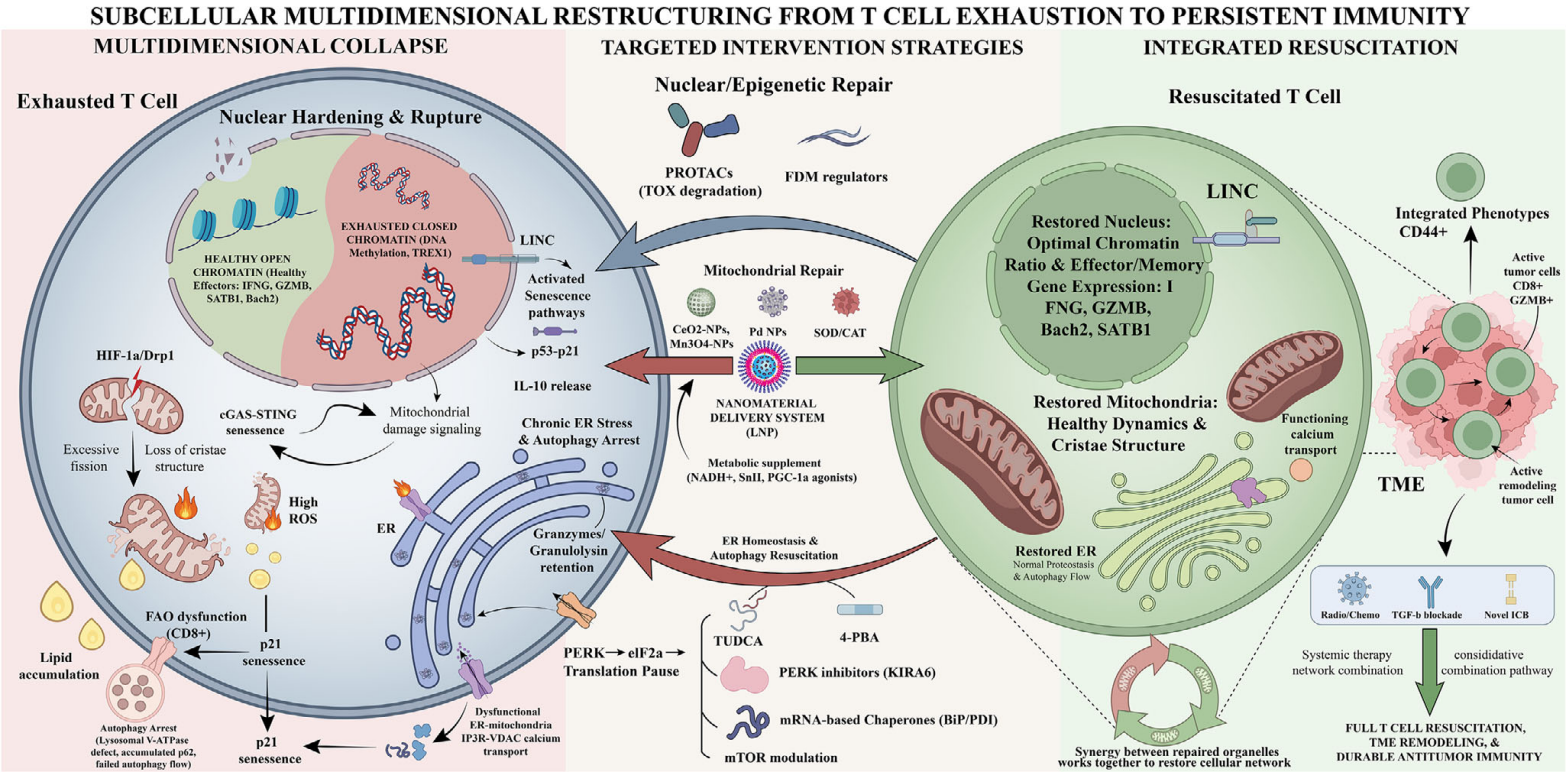
Subcellular multidimensional collapse and integrated reinvigoration strategies of Tex.

Over the past decade, the emergence of ICB therapy has profoundly transformed the paradigm of oncology treatment, marking a conceptual shift from directly targeting tumor cells to systemically mobilizing host immune responses. By blocking inhibitory signaling pathways such as cytotoxic T‐lymphocyte‐associated protein 4 (CTLA‐4) and programmed cell death protein 1/programmed death‐ligand 1 (PD‐1/PD‐L1), this therapy aims to restore the effector functions of tumor‐infiltrating lymphocytes (TILs), demonstrating the potential to prolong patient survival in certain advanced malignancies, including melanoma and non‐small cell lung cancer (NSCLC) [1, 2]. However, its clinical application still faces significant limitations. In unselected patients with solid tumors, the objective response rate (ORR) of ICB monotherapy typically ranges from merely 15% to 30% [3, 4]. For tumor types characterized by sparse immune infiltration, such as pancreatic cancer and glioblastoma, their dense TME frequently results in primary resistance [5, 6]. Furthermore, even in initially responding patients, the progressive decline of T‐cell function over the course of treatment frequently leads to acquired resistance [7]. These clinical challenges indicate that merely blocking cell surface inhibitory signals is insufficient to overcome the complex immunosuppressive networks within solid tumors. Consequently, an in‐depth exploration of the fundamental mechanisms underlying T‐cell dysfunction, particularly the highly heterogeneous processes Figure 1 of adaptation and exhaustion within the hostile TME, has emerged as a core issue urgently requiring resolution in the field.

Traditionally, Tex has been regarded as a generic differentiation state characterized by the loss of effector functions and the sustained high expression of inhibitory receptors [8]. Subsequent studies further revealed that this state is driven by key transcription factors, such as thymocyte selection‐associated high mobility group box (TOX), nuclear receptor subfamily 4 group A (NR4A), and nuclear factor of activated T cells (NFAT), and is accompanied by specific chromatin remodeling, culminating in a stable epigenetic landscape distinct from that of effector or memory T cells [9, 10]. However, recent advances underscore that exhaustion is not a singular endpoint, but a continuous spectrum characterized by profound lineage and clonotypic heterogeneity. Mounting evidence indicates that the subcellular metabolic requirements and organellar homeostasis differ fundamentally between progenitor exhausted subsets (Tpex) that retain stemness and terminally exhausted subsets (Terminal Tex) that suffer severe functional decay. During the exhaustion process, beyond phenotypic alterations, profound pathological modifications occur within internal subcellular structures, including mitochondria, the ER, lysosomes, and the nucleus [11]. Therefore, this review explores the association between terminal exhaustion and systemic suborganellar dysfunction, proposing that persistent perturbations at this subcellular level, rather than mere surface receptor expression, represent a major biological barrier to ICB efficacy.

The fundamental drivers of this subcellular remodeling originate from the TME. First, aberrant tumor vasculature induces tissue hypoxia and glucose deprivation, directly impairing mitochondrial energy metabolism in T cells and compelling them to undergo metabolic reprogramming [12, 13]. Second, the robust glycolysis of tumor cells results in a massive accumulation of lactic acid; the subsequent acidification of the microenvironment disrupts the pH homeostasis of T‐cell lysosomes, thereby compromising their degradative capacity [14]. Furthermore, recent studies have highlighted the role of the physical microenvironment: the highly fibrotic stroma significantly elevates tissue stiffness, subjecting migrating T cells to continuous mechanical stress. This can precipitate nuclear deformation and even compromise nuclear envelope integrity, thereby triggering genomic instability [15]. The synergistic effects of these multiple TME stressors ultimately drive distinct T‐cell subpopulations toward divergent fates, forcing resilient subsets to undergo adaptive remodeling while driving the bulk effector compartment into terminal organellar collapse.

Building upon these premises, this review aims to re‐evaluate the mechanisms of Tex from a subcellular organelle perspective. We systematically elucidate the dysregulation of organelle crosstalk networks under TME stress, prominently highlighting the lineage, temporal, and clonotypic heterogeneity that dictates whether a T cell maintains progenitor‐like stemness or plunges into terminal organellar collapse. Furthermore, we summarize the specific spatial patterns of organelle damage across diverse solid tumor niches. Finally, this article reviews emerging, primarily preclinical engineering strategies targeting these subcellular checkpoints. While exploring conceptual innovations such as nanoparticle‐mediated metabolic modulation and synthetic biology, we explicitly delineate the current gap between mechanistic plausibility in preclinical models and clinically actionable interventions, aiming to provide objective insights for the development of next‐generation precision immunotherapy.

## 2 Biological Basis of T‐Cell Exhaustion in the Tumor Microenvironment

### 2.1 Intrinsic and Extrinsic Drivers of T‐Cell Suppression

Within the TME, the subcellular structure and function of T cells are severely disrupted, stemming from the aberrant activation of cell‐intrinsic regulatory programs and their continuous interaction with extrinsic immunosuppressive networks. The core mechanism of intrinsic T‐cell inhibition is characterized by the sustained upregulation of co‐inhibitory receptors, such as PD‐1, CTLA‐4, T‐cell immunoglobulin and mucin‐domain containing‐3 (TIM‐3), and lymphocyte‐activation gene 3 (LAG‐3). These receptors serve not only as phenotypic markers of T‐cell exhaustion (Tex) but also as active signal transduction and metabolic brakes [16]. For instance, the ligation of PD‐1 suppresses signaling cascades downstream of the proximal T‐cell receptor (TCR) and CD28, thereby significantly impairing T‐cell activation. Furthermore, persistent co‐inhibitory signaling enforces profound metabolic reprogramming, restricting glycolysis and fatty acid oxidation pathways, which markedly diminishes the bioenergetic capacity required to sustain durable effector functions [17].

Beyond these cell‐intrinsic defects, Tex is significantly influenced by a dense network of immunosuppressive cells within the TME. A prominent extrinsic factor is the dynamic polarization of macrophages toward an M2‐like phenotype. In diverse malignant contexts, including breast cancer, glioblastoma, and colorectal cancer, these M2 macrophages shape a local immunosuppressive milieu through the secretion of inhibitory cytokines, such as transforming growth factor‐beta (TGF‐β) and interleukin‐10 (IL‐10), along with various metabolic inhibitors [18]. Concurrently, regulatory T cells (Tregs) and myeloid‐derived suppressor cells (MDSCs) exert potent extrinsic inhibition through metabolic competition, the generation of reactive oxygen species (ROS), and the continuous depletion of essential amino acids, collectively driving effector T cells into a state of metabolic deprivation [19]. Notably, CD71+ erythroid cells (CECs) have recently been identified as critical extrinsic mediators of both systemic and local immunosuppression. Initially recognized for their regulatory properties in neonatal immune tolerance, these cells are now found to be highly enriched in various pathological conditions, including advanced solid tumors [20, 21]. Substantial evidence indicates that CECs exert robust extrinsic inhibition on T‐cell effector functions through mechanisms involving ROS production and arginase‐2 activity. Their pathological expansion not only impairs intrinsic T‐cell immune responses but also serves as a predictive biomarker for poor prognosis following immune checkpoint inhibitor (ICI) therapy, underscoring their indispensable role within the extrinsic inhibitory network.

### 2.2 Characteristics of Tex in the TME

In the TME of solid tumors, Tex is a differentiation state driven by persistent antigen exposure, featuring unique transcriptional and epigenetic profiles [22]. Unlike memory T cells generated following acute immune responses, exhausted T cells undergo a structured hierarchical differentiation process. This lineage begins with TCF‐1‐expressing Tpex, which retain stem‐like self‐renewal capacity and the primary potential to mount a proliferative burst in response to immune checkpoint blockade (ICB). Under persistent antigen stimulation, these progenitors undergo unidirectional epigenetic and transcriptional reprogramming, transitioning into terminally exhausted T cells (Tex). This terminal state is characterized by the permanent loss of effector function, the high co‐expression of inhibitory receptors (e.g., PD‐1, TIM‐3, LAG‐3), and the sustained upregulation of the master regulator TOX [23, 24, 25, 26, 27]. The core molecular mechanism lies in the fact that persistent TCR signaling within the TME leads to the continuous activation of the transcription factor NFAT by calcineurin. In the absence of synergistic cooperation from the activator protein‐1 (AP‐1) transcription factor complex, NFAT alone initiates an aberrant transcriptional program, subsequently upregulating the expression of exhaustion‐associated genes such as TOX, NR4A, and programmed cell death protein 1 gene (PDCD1) [28, 29]. Among these, the TOX protein, acting as a crucial chromatin remodeling factor, recruits histone deacetylases and DNA methyltransferases to enforce durable epigenetic silencing at the loci of effector genes such as interferon‐gamma gene (IFNG) and granzyme B gene (GZMB) [30]. This solidified, aberrant epigenetic program is a major factor contributing to the profound resistance of terminally exhausted T cells to ICB therapy. Although PD‐1/PD‐L1 blockade can transiently restore downstream TCR signaling, it fails to reverse the stably established inhibitory modifications at the genomic level, rendering it exceedingly difficult for T cells to regain durable and potent effector functions [31].

Conventional wisdom posits that these epigenetic and organellar failures occur as parallel events. However, a definitive hierarchical causal relationship exists between the two. Early organellar dysfunction, specifically mitochondrial ROS burst and ER calcium leakage, serves as the initial metabolic trigger, activating the calcineurin, NFAT, and TOX signaling axis. This signaling cascade initiates the epigenetic locking process. Conversely, once the epigenetic landscape becomes fixed, it transcriptionally suppresses organellar biogenesis, for instance by silencing PGC1A, thereby converting early functional stress into irreversible structural collapse. This feed‐forward loop renders T cell exhaustion not merely a signaling state but a final metabolic and epigenetic fate.

### 2.3 Organelle Dysfunction in the TME

The functional decline of T cells within the tumor microenvironment exhibits profound lineage and temporal heterogeneity. Exhaustion is not a singular physiological endpoint but rather a continuous differentiation spectrum. Distinct T cell subsets display fundamental differences in subcellular metabolic demands and organellar homeostasis. Progenitor exhausted CD8^+^ T cells, which are essential for sustaining durable antitumor immunity, stringently preserve mitochondrial fatty acid oxidation and OXPHOS capacity to maintain stemness and self‐renewal potential [32]. Conversely, terminally exhausted T cells, which constitute the majority of tumor‐infiltrating lymphocytes, predominantly undergo severe physical structural and metabolic collapse of organelles. Furthermore, Tregs and TRM cells acquire significant survival advantages within the hypoxic and nutrient‐deprived microenvironment through specific organellar remodeling [33]. Therefore, the subsequent discussion regarding subcellular structural and functional perturbations will primarily focus on the terminally exhausted T cell population that drives the overall immunosuppressive phenotype, while also elucidating the heterogeneous organellar fates exhibited by T cells of distinct differentiation states and clonotypes subjected to the same microenvironmental pressures. As the energy hub, mitochondria experience dynamic perturbations and respiratory chain anomalies that can substantially impair bioenergetics, thereby compromising the energy supply requisite for effector functions [34]. Concurrently, the imbalance in the protein synthesis and degradation network constituted by the ER and lysosomes provokes a sustained unfolded protein response (UPR) and the accumulation of metabolic waste, thereby impeding the secretion of critical effector proteins [35, 36]. Furthermore, the nucleus, serving as the center for mechanosensation and genetic information, is susceptible to nuclear envelope damage and genomic instability under the physical compression of the dense stroma, which subsequently drives the cell into a state of senescence [37, 38].

Therefore, elucidating Tex from a subcellular organelle perspective unveils the profound mechanisms underlying resistance to ICB therapy. When core intracellular functions sustain substantial impairment, thereby disrupting bioenergetic processes, proteostasis, and genomic integrity, merely blocking surface inhibitory signals is insufficient to restore the comprehensive effector functions of T cells.

### 2.4 Classical Exhaustion versus Non‐Exhaustion T Cell Dysfunction

The functional decline of T cells within the tumor microenvironment is highly heterogeneous. Mechanistically distinguishing classical T cell exhaustion from other stress‐induced dysfunctional states is essential, as the latter represent distinct cellular outcomes driven by divergent subcellular events.

**FIGURE 2:**
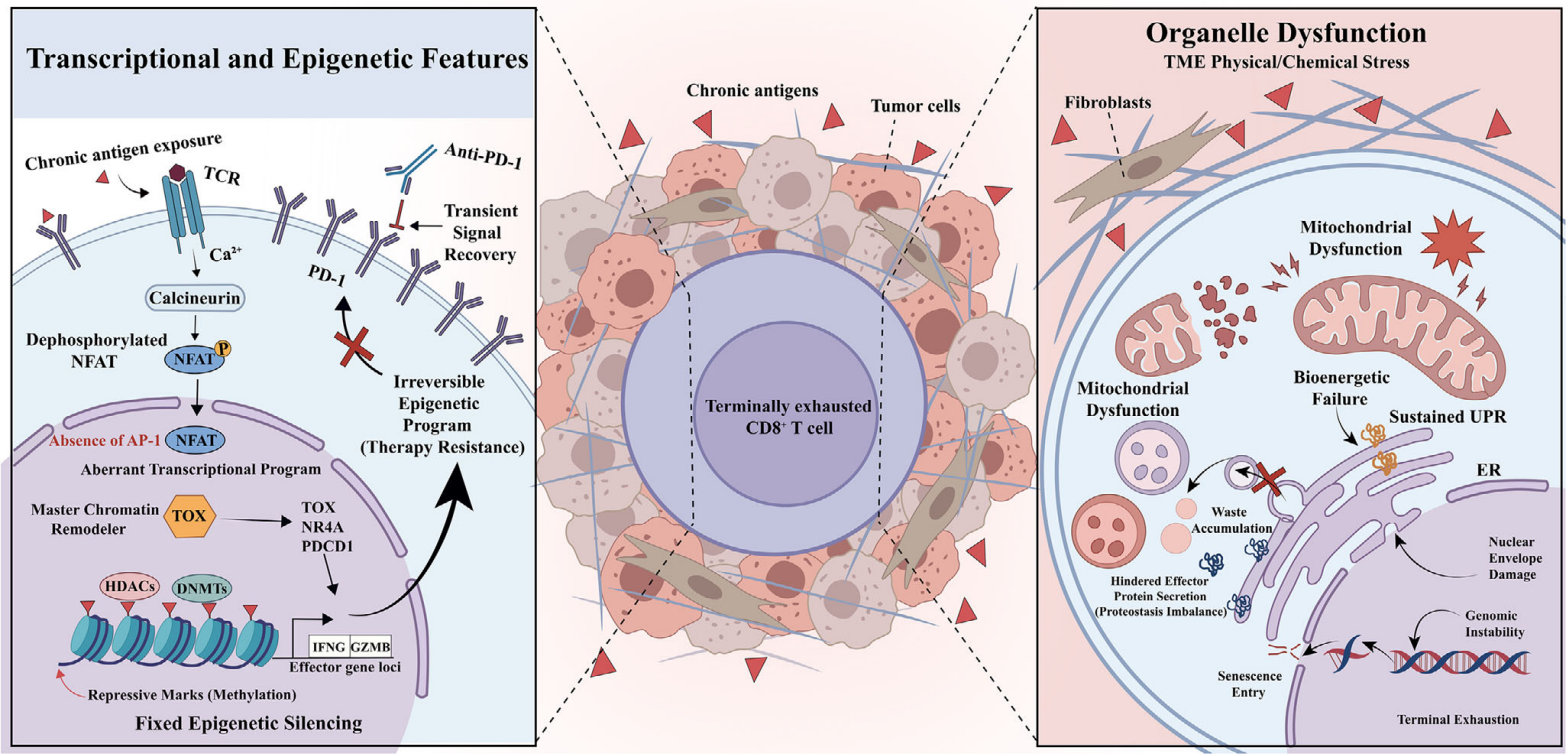
Dual driving mechanisms of tex in the TME: epigenetic remodeling and organelle damage.

1. The Classical Exhaustion Program: This state is primarily driven by persistent antigen exposure and is coordinately regulated by transcription factors including TOX and the NR4A family [10, 39]. Its molecular hallmarks include stable repressive epigenetic modifications and sustained high‐level expression of coinhibitory receptors such as PD‐1, TIM‐3, and LAG‐3. Distinct from terminal differentiation or senescence, classically exhausted T cells can survive and persist long‐term within the tumor microenvironment, yet they exhibit progressive and hierarchical attrition of proximal TCR signaling and effector function.
2. Bioenergetic Collapse‐Induced Terminal Dysfunction: This state is distinct from epigenetically driven exhaustion and occurs under conditions of extreme metabolic stress, such as severe hypoxia, profound glucose deprivation, or substrate deficiency for mitochondrial OXPHOS [40]. Under these circumstances, mitochondrial respiratory chain supercomplexes undergo physical disassembly, and ATP synthetic capacity declines precipitously. Even if the cell retains a degree of transcriptional competence, the fundamental collapse of bioenergetic supply renders the T cell physically incapable of sustaining effector function, manifesting as irreversible functional loss [41].
3. Senescence‐Like Dysfunction: This trajectory is typically triggered by persistent DNA damage or chronic innate immune signaling, the former exemplified by genomic instability consequent to nuclear envelope rupture and the latter by cGAS‐STING pathway activation following mitochondrial DNA leakage [42, 43]. Unlike classical exhaustion, senescence‐like T cells enter irreversible cell cycle arrest and acquire a senescence‐associated secretory phenotype, further reinforcing the local immunosuppressive microenvironment through secretion of cytokines including IL‐10, TGF‐β, and IL‐6.
4. Activation‐Induced Cell Death and Ferroptosis: When T cells encounter severe organellar stress, such as excessive activation of lipid peroxidation or disruption of ER calcium homeostasis, they do not persist in a functionally suppressed state but are instead physically eliminated via apoptosis or ferroptosis pathways [44, 45]. This process results in an absolute reduction of the intratumoral T cell pool, a cardinal distinction from the long‐term persistence of exhausted cells, and plays a unique role in resistance to immunotherapy (Figure 2).

## 3 Mitochondrial Dysfunction and Metabolic Remodeling in the TME

### 3.1 Abnormal Mitochondrial Dynamics

The dynamic remodeling of mitochondrial morphology represents not merely an alteration in organelle appearance, but rather a core metabolic checkpoint for T cells adapting to TME stress [46]. In effector T cells (Teffs), the rapid cycling of mitochondrial fusion and fission provides a highly efficient ATP supply for the formation and function of immune synapses [47, 48]. However, in Terminal Tex, this dynamic equilibrium is significantly disrupted, manifesting as an aberrant state dominated by excessive fission and accompanied by impaired fusion mechanisms. This mitochondrial structural remodeling directly impacts their core functions, leading to the abnormal assembly and dysfunction of electron transport chain (ETC) complexes, and precipitating the excessive accumulation of ROS [49].

#### 3.1.1 Mitochondrial Fission under Hypoxic Conditions

Within the complex TME of solid tumors, severe hypoxia serves as a critical signal driving the dysfunctional remodeling of T‐cell mitochondria, profoundly impacting the durability of anti‐tumor immune responses [50]. While moderate mitochondrial fission facilitates the clonal expansion of Teffs during the activation phase, within tumor tissues, the persistently stabilized hypoxia‐inducible factor 1‐alpha (HIF‐1α) hyperactivates the fission protein dynamin‐related protein 1 (Drp1) via the CaMKII‐ extracellular signal‐regulated kinase 1/2 (ERK1/2) signaling axis, leading to an excessively fragmented mitochondrial state [51, 52]. This structural alteration directly impairs mitochondrial function; fragmented mitochondria struggle to maintain normal membrane potential, precipitating the excessive accumulation of ROS. This process selectively attenuates the motility of Terminal Tex, restricting their infiltration into the deep tumor bed and curtailing their survival via apoptosis. In contrast, Tpex and Tregs often resist this extreme fragmentation, maintaining sufficient mitochondrial fitness for prolonged TME residency. Therefore, TME‐induced excessive mitochondrial fission acts as a potential defensive mechanism employed by tumor cells to promote immune cell exhaustion metabolically, providing a structural framework for the rapid depletion of T cells within the tumor.

#### 3.1.2 Downregulation of Fusion Proteins and Abnormal Cristae Structure

Simultaneously, the intense nutritional competition between tumor cells and T cells directly disrupts the mitochondrial fusion mechanism, thereby attenuating the cytotoxic function of T cells. To satisfy their requirements for rapid proliferation, tumor cells massively deplete critical amino acids, including essential and conditionally essential species such as tryptophan, arginine, cysteine, and glutamine, within the TME [53]. Upon sensing these nutrient‐deprivation signals, T cells activate the mitochondrial metalloprotease OMA1, leading to the proteolytic cleavage and subsequent inactivation of the critical fusion protein optic atrophy 1 (Opa1) [54].

The loss of Opa1 induces a loose and disorganized mitochondrial cristae architecture, disrupting the proper assembly and spatial conformation of respiratory chain supercomplexes [55, 56]. Given that the synthesis, processing, and secretion of cytotoxic proteins, such as perforin and granzymes, are highly energy‐demanding processes, the perturbation of cristae structure directly diminishes the efficiency of oxidative phosphorylation (OXPHOS), resulting in an inadequate energy supply [57, 58]. A study by Buck et al. demonstrated that in a melanoma model, the downregulation of Opa1 in T cells engenders a dysfunctional state wherein the cells can recognize tumor antigens but fail to effectively secrete granzymes due to defective energy metabolism, thereby constituting a “recognize but cannot kill” phenotype [56]. This mechanism is likely a downstream consequence that facilitates tumor immune evasion.

#### 3.1.3 Inhibition of Mitochondrial Biogenesis

Furthermore, unlike acute viral infection, chronic antigen stimulation in the TME leads to stagnation of mitochondrial turnover, a phenomenon closely associated with resistance to ICB therapy [59, 60]. Under persistent antigen exposure, the expression of the transcriptional repressor B lymphocyte‐induced maturation protein‐1 (Blimp‐1) is upregulated in T cells, which subsequently markedly suppresses peroxisome proliferator‐activated receptor gamma coactivator 1‐alpha (PGC1α), the master regulator of mitochondrial biogenesis [40, 61]. This mechanism may constitute a potential factor underlying the poor efficacy of anti‐PD‐1 therapy; ICB therapy aims to restore T cell function by blocking inhibitory signals, but this requires T cells to possess the capacity for rapid synthesis of new mitochondria to support their functional recovery and proliferation. However, under Blimp‐1‐mediated suppression, terminally exhausted T cells—particularly those derived from high‐affinity clonotypes—are replete with senescent and dysfunctional mitochondria, lacking the capability to generate new organelles [62]. Therefore, merely blocking the PD‐1 signaling pathway primarily drives the expansion of Tpex cells, but is insufficient to restore the effector functions of the bulk Terminal Tex population, as their internal mitochondrial metabolic machinery has undergone irreversible decline. Epigenetic silencing of the mitochondrial biogenesis program constitutes a key metabolic checkpoint in terminal Tex and is closely associated with primary or acquired resistance to anti‐PD‐1 immunotherapy.

### 3.2 Abnormal Metabolic Reprogramming

#### 3.2.1 Decline in Oxidative Phosphorylation Efficiency

In the TME, the anti‐tumor effector functions of T cells are exceptionally energy‐demanding processes heavily reliant on ATP, encompassing cytokine synthesis, granzyme secretion, and chemotactic migration deep into the tumor bed [63]. However, the Terminal Tex compartment exhibits profoundly impaired oxidative phosphorylation, a process further exacerbated by structural disruption of the supramolecular assembly of the mitochondrial electron transport chain [55]. Conversely, tumor‐infiltrating Tregs frequently preserve intact supercomplex architectures to sustain robust OXPHOS. Mechanistically, the hypermetabolism of tumor cells depletes glucose and oxygen within the microenvironment; this metabolic stress compromises the integrity of the inner mitochondrial membrane and cristae architecture [64]. Under physiological conditions, ETC complexes I, III, and IV assemble into highly efficient respiratory supercomplexes to facilitate synergistic electron transfer. Nevertheless, under TME stress, mitochondrial cristae remodeling precipitates the dissociation of these supercomplexes, severely impairing the functional coupling among them [65, 66]. This structural dissociation significantly diminishes electron transfer efficiency and ATP synthesis capacity; consequently, due to ATP insufficiency, T cells fail to effectively drive actin cytoskeletal rearrangement, thereby attenuating their capacity to form stable immune synapses with target cells and severely limiting the efficacy of contact‐dependent cytotoxic mechanisms.

#### 3.2.2 ROS Accumulation and Oxidative Damage

The abnormal assembly of the ETC further triggers a cascade reaction, wherein electrons aberrantly leak from the uncoupled complexes I and III and react with molecular oxygen to generate excessive ROS, such as superoxide anions [67, 68]. Physiological levels of mitochondrial ROS serve as requisite signaling molecules for T‐cell activation; however, within the TME, continuous hypoxia‐reoxygenation cycles cause the generation rate of ROS to outpace the clearance capacity of cellular antioxidant systems. This disruption of redox homeostasis inflicts extensive molecular damage [17, 69]. At the lipid level, excessive ROS attack polyunsaturated fatty acids (PUFAs) on the mitochondrial membrane, initiating lipid peroxidation. This not only alters the physical properties of the membrane but also depletes the critical antioxidant enzyme glutathione peroxidase 4 (GPX4), thereby selectively exacerbating the susceptibility of terminally exhausted effector T cells to ferroptosis [70, 71, 72]. At the protein level, oxidative stress can induce inactivating carbonylation modifications on pivotal metabolic enzymes and cytoskeletal proteins [73, 74]. Ultimately, persistent oxidative stress, through mechanisms such as activating the DNA damage response or extreme lipid peroxidation, pushes T cells toward a senescence‐like state or programmed cell death, respectively, thereby accelerating T‐cell functional decline and impairing the persistence of functional T cells within the tumor [75, 76].

#### 3.2.3 Fatty Acid Oxidation Dysfunction and Subset‐Specific Metabolic Reprogramming

When glucose is severely restricted within the TME, T cells attempt to initiate metabolic reprogramming from glycolysis toward FAO. However, this metabolic switch exhibits profound subset specificity. For Tpex cells, maintenance of a highly fused mitochondrial network and optimal membrane potential enables efficient utilization of FAO and OXPHOS, thereby preserving multipotency and responsiveness to immune checkpoint blockade [77, 78]. As the differentiation trajectory advances toward terminal exhaustion, however, this metabolic flexibility is substantially lost. Driven concurrently by chronic TCR overstimulation and hypoxia, terminally exhausted T cells manifest severe uncoupling of respiratory supercomplexes and disruption of mitochondrial cristae architecture [79]. This structural deterioration renders their passive reliance on FAO incapable of generating sufficient ATP supply; instead, oxidized lipids aberrantly imported via CD36 further exacerbate mitochondrial lipid peroxidation, trapping Terminal Tex cells in irreversible bioenergetic collapse [80, 81]. In contrast, tumor‐infiltrating Tregs exploit this metabolic axis to gain a selective advantage. These cells circumvent the mitochondrial fragmentation that afflicts terminally exhausted T cells and sustain robust OXPHOS and FAO efficiency within the low‐glucose milieu. Such subcellular adaptation endows them with enhanced capacity for scavenging free fatty acids from the microenvironment, thereby potentially restricting energy substrate availability for adjacent effector T cell populations.

### 3.3 Abnormal Mitochondria‐Associated Immune Signaling

#### 3.3.1 Cytosolic Release of mtDNA

Under physiological conditions, mitochondrial DNA (mtDNA) within Teffs is strictly confined to the mitochondrial matrix to avert the inappropriate activation of cytosolic innate immune signaling [82]. However, within the solid TME, this compartmentalization is disrupted, thereby precipitating T‐cell dysfunction [79].

The synergistic interplay between high oxidative stress and calcium homeostasis imbalance within tumor tissues induces conformational changes in the adenine nucleotide translocator (ANT) on the inner mitochondrial membrane and the voltage‐dependent anion channel (VDAC) on the outer membrane, consequently forming a highly permeable mitochondrial permeability transition pore (mPTP) [83, 84]. Concurrently, the hyperactivated fission protein Drp1 and the pro‐apoptotic proteins Bax/Bak oligomerize on the outer mitochondrial membrane, further compromising membrane integrity and providing a conduit for the release of mtDNA [85, 86]. These structural alterations result in the continuous leakage of mtDNA into the cytoplasm. Rich in unmethylated CpG motifs, mtDNA functions as an endogenous danger signal molecule. Its aberrant accumulation in the cytosol inappropriately activates cytosolic nucleic acid sensing pathways—which are physiologically intended for pathogen recognition—thereby potentially interfering with the specific recognition and response of T cells to tumor antigens [87].

#### 3.3.2 Sustained Activation of the cGAS‐STING Signaling Axis

The aberrantly accumulated mtDNA in the cytosol is recognized by the DNA sensor cyclic GMP‐AMP synthase (cGAS), which subsequently activates its downstream STING signaling pathway. During acute immune responses, transient STING activation promotes T‐cell expansion and enhances antigen presentation via the induction of type I interferons (IFNs) [88]. However, within the TME, the cGAS‐STING signaling elicited by the continuous leakage of mtDNA manifests as a chronic, low‐intensity sustained activation state; this aberrant persistence significantly alters the downstream output of the pathway [89]. Rather than effectively driving anti‐tumor effector programs, it is predisposed to inducing the expression of immunosuppressive cytokines, prototypically IL‐10 and TGF‐β [62]. More importantly, chronic STING signaling can trigger p53‐p21‐dependent cell cycle arrest, driving T cells into a permanent senescence‐like state. Unlike exhausted T cells, which may retain residual proliferative potential, these senescent cells undergo irreversible growth arrest while acquiring enhanced anti‐apoptotic capacity and an inhibitory secretory phenotype [42]. They persist for extended periods within the TME in a metabolically active yet functionally silent form; consequently, they not only compete for limited survival space and resources but may also further suppress the functions of surrounding Teffs via the secretion of a milieu of factors.

#### 3.3.3 Defective Mitophagy

**FIGURE 3:**
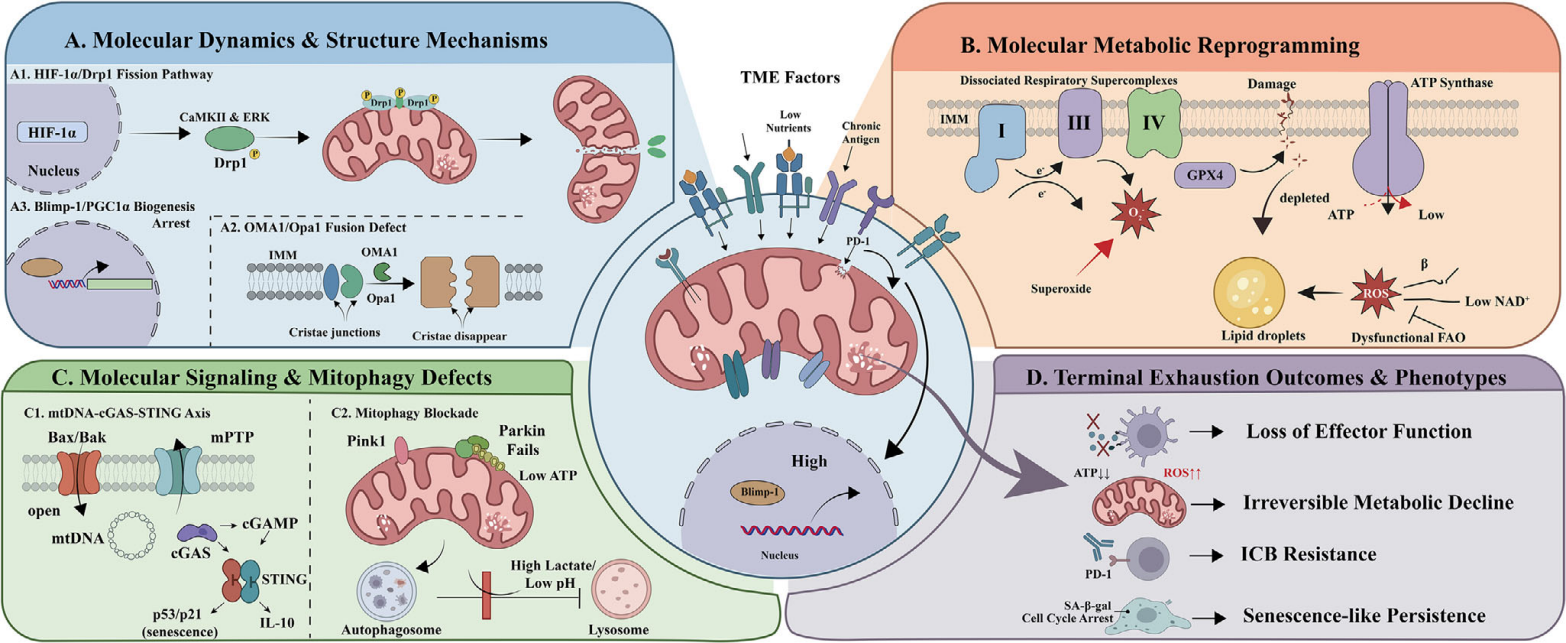
Multidimensional collapse of exhausted T cells at the mitochondrial level: structural dynamic imbalance, metabolic reprogramming, and autophagic defects.

To maintain cellular homeostasis, impaired mitochondria are typically eliminated via the mitophagy pathway. However, upon transition to the Terminal Tex, this critical quality control process exhibits significant impediments, resulting in the persistent accumulation of dysfunctional mitochondria [79, 90]. In contrast, Tpex cells maintain relatively intact autophagic flux to clear damaged organelles. From a molecular mechanistic perspective, severe hypoxia and the loss of mitochondrial membrane potential within the TME ought to activate the stable accumulation of the PTEN‐induced kinase 1 (PINK1) on the outer mitochondrial membrane, subsequently recruiting the E3 ubiquitin ligase Parkin [91, 92]. Nevertheless, within Terminal Tex, the diminished ATP levels compromise the efficiency of subsequent ubiquitin chain assembly; concomitantly, the highly lactic acid‐enriched environment impedes the fusion of autophagosomes with lysosomes [93]. This engenders a self‐reinforcing negative feedback loop wherein dysfunctional mitochondria cannot be effectively cleared; instead, they continuously generate ROS and leak mtDNA, thereby perpetually exacerbating the aforementioned immune signaling perturbations [94]. This abrogation of autophagic capacity precipitates the massive accumulation of dysfunctional organelles within Terminal Tex. This not only diminishes cellular responsiveness to anti‐PD‐1 blockade therapy but also confines the cells to a terminal state characterized by a loss of effector functions and restricted self‐renewal capacity. Therefore, the failure of mitophagy could contribute to the compromised ability of T cells to recover their functions within the TME (Figure 3).

## 4 Homeostatic Imbalance of the ER and Lysosomes in the TME

The ER and lysosomes collaboratively constitute the core organelle network that maintains proteostasis in T cells, orchestrating the synthesis, folding, modification, and degradation of effector proteins [95]. During the phases of T‐cell activation and expansion, to fulfill the requisites of effector functions, the secretion of cytokines (e.g., interferon‐gamma [IFN‐γ] and tumor necrosis factor‐alpha [TNF‐α]) alongside the assembly of cytotoxic granules escalates dramatically, imposing extraordinarily high demands on the biosynthetic and folding capacities of the ER [96]. However, within the solid TME, the contradiction between the deprivation of metabolic substrates and the vigorous biosynthetic demands of T cells abrogates the functional coordination between the ER and lysosomes. Terminal Tex typically manifest a sustained, aberrant activation of the UPR, wherein its signaling output transitions from physiological adaptive repair to driving pro‐apoptotic pathways and functional inhibition [35];concomitantly, the degradative capacity of lysosomes is markedly compromised. This marked dysregulation of the protein homeostasis network is not only a direct effect of metabolic stress on T cells but also an important factor limiting their antitumor efficacy and leading to resistance to ICB therapy.

### 4.1 ER Stress and the UPR

#### 4.1.1 Functional Shift of the IRE1α‐XBP1 Signaling Axis

The profound glucose deprivation within the TME constitutes a critical initiating factor for ER stress, particularly in highly proliferative effector subsets and Terminal Tex burdened with massive secretory demands. Because N‐linked glycosylation of proteins is highly glucose‐dependent, the insufficiency of its substrates leads to the misfolding of nascent proteins within the ER lumen, thereby activating inositol‐requiring enzyme 1 alpha (IRE1α), the core sensor of the UPR [97]. Under acute stress, activated IRE1α cleaves X‐box binding protein 1 (XBP1s), mRNA to generate the transcriptionally active XBP1s protein, which subsequently upregulates the expression of molecular chaperones to enhance the folding capacity of the ER; this process is pivotal for the differentiation of Teffs [98]. However, under the chronic metabolic stress prevalent in solid tumors such as ovarian cancer and melanoma, IRE1α enters a state of sustained activation. In this persistently activated state, the endonuclease activity of IRE1α undergoes reprogramming, shifting from predominantly mediating XBP1 splicing to initiating the regulated IRE1‐dependent decay (RIDD) pathway. The RIDD program extensively degrades the mRNAs of diverse functional proteins, including critical TCR subunits and anti‐apoptotic factors, thereby attenuating TCR signal transduction and significantly diminishing the sensitivity of T cells in recognizing and responding to tumor antigens [99]. Furthermore, a study by Song et al. revealed another facet of this axis: the chronically accumulated XBP1s can directly bind to and transactivate the promoter of the PDCD1 gene, thereby forging a positive feedback loop at the transcriptional level that sustains the state of Tex [98].

#### 4.1.2 Lineage‐Dependent Outcomes of the PERK‐eIF2α‐ATF4 Axis

Amino acid deprivation and ROS accumulation within the TME cooperatively activate the PERK‐eIF2α‐ATF4 signaling arm of the UPR. Critically, the subcellular consequences of this ER stress response are strictly governed by T cell lineage identity and activation history. In terminally exhausted T cells subjected to persistent antigen stimulation, excessive demand for effector protein synthesis vastly exceeds ER folding capacity [100]. This unmitigated ER stress results in hyperactivation of the PERK‐ATF4‐CHOP apoptotic axis. Sustained CHOP induction not only suppresses the critical transcription factor T‐bet but also directly drives Terminal Tex cells toward proteotoxic dysfunction and mitochondrial collapse [101, 102]. Conversely, the ER stress pathway is repurposed as a critical adaptive survival mechanism in immunosuppressive subsets. Through fine‐tuned modulation of UPR sensors such as PERK, Tregs effectively buffer lipotoxicity and hypoxic stress within the microenvironment [103]. This controlled ER stress response functions as a protective barrier, facilitating the sustained high‐throughput synthesis and secretion of suppressive cytokines including TGF‐β and IL‐10 without precipitating lethal ER collapse. Thus, ER stress manifests as a terminal execution mechanism in antigen‐overloaded Terminal Tex cells, whereas in immunosuppressive subsets it serves as a core driver for maintaining subcellular homeostasis and microenvironmental adaptation.

#### 4.1.3 Dysregulation of the ATF6 Signaling Axis

Compared to other branches of the UPR, the role of the activating transcription factor 6 (ATF6) pathway in Tex has not been fully appreciated; it actually represents a critical adaptive mechanism for cells contending with TME stress [104]. In Teffs, activated ATF6 translocates to the Golgi apparatus for proteolytic cleavage, releasing its cytosolic fragment to the nucleus where it functions as a transcription factor to predominantly upregulate the expression of ER chaperones, such as BiP (GRP78) and GRP94; this is indispensable for ensuring the proper folding and assembly of effector proteins, including cytotoxic granules [105]. However, in Terminal Tex from advanced‐stage tumors, the adaptive functionality of the ATF6 signaling pathway is significantly attenuated, whereas Tregs can often harness this pathway to sustain suppressive proteostasis. This is primarily attributed to TME‐induced alterations in ER membrane lipid composition, which impede the vesicular transport of ATF6 from the ER to the Golgi, thereby precluding its effective activation [106]. The insufficient synthesis of chaperones results in an inability to timely process misfolded proteins, which subsequently form insoluble aggregates within the cell. The formation of these aggregates not only disrupts intracellular trafficking but may also activate the caspase‐8‐mediated apoptotic pathway [107]. Therefore, the failure of the ATF6 signaling axis signifies a loss of the critical capacity of T cells to maintain proteostasis, rendering them more susceptible to dysfunction under persistent antigenic stimulation. This also constitutes a potential mechanism limiting the efficacy and persistence of adoptive cell therapy (ACT) in solid tumors.

### 4.2 Alterations in Lipid Metabolism and Membrane Function

#### 4.2.1 Dysregulation of Cholesterol Homeostasis

In TMEs characterized by highly active lipid metabolism, such as hepatocellular carcinoma (HCC) and breast cancer, T cells frequently encounter the challenge of aberrant lipid accumulation; this metabolic state suppresses effector functions by interfering with cholesterol homeostasis. Cholesterol is not only a fundamental structural component of cell membranes but also constitutes the core of lipid rafts, which serve as critical platforms for TCR signal transduction [108]. The Terminal Tex population exhibits significant perturbations in cholesterol metabolism, one hallmark of which is the upregulation of acyl‐CoA: cholesterol acyltransferase 1 (ACAT1). ACAT1 catalyzes the esterification of free cholesterol into cholesterol esters, which are subsequently sequestered within cytosolic lipid droplets. This process depletes the free cholesterol pool on the plasma membrane, disrupting the membrane environment requisite for the assembly of TCR signaling complexes [109]. Research indicates that oxidized low‐density lipoprotein (oxLDL) within the TME, upon uptake via the CD36 receptor, can activate the PPAR‐γ signaling axis, thereby driving the aberrant expression of ACAT1. The esterified, sequestered cholesterol is unavailable for the formation of the immune synapse, leading to diminished sensitivity of CD8^+^ T cells toward tumor antigens [110, 111]. Notably, pharmacological inhibition or genetic knockdown of ACAT1 can reduce cholesterol esterification and elevate plasma membrane free cholesterol levels, thereby promoting TCR signaling and restoring T‐cell proliferation and cytotoxic function; this suggests that ACAT1 is a significant potential target for reversing metabolic insufficiency in T cells.

Furthermore, beyond direct targeting of intracellular lipid esterification, modulating systemic and microenvironmental cholesterol levels using clinically available agents has emerged as a strategy with translational potential. Emerging evidence indicates that cholesterol‐lowering drugs can regulate the fate of tumor‐infiltrating CD8^+^ T cells. By reducing cholesterol accessibility within the TME, such agents effectively downregulate the expression of key coinhibitory receptors on CD8^+^ T cells, thereby attenuating the severity of CD8^+^ T cell exhaustion [112]. This suggests that repurposing widely prescribed cholesterol‐lowering medications may serve as a valuable pharmacological adjunct. When combined with immune checkpoint blockade, such interventions could help prevent the consolidation of a lipid‐driven exhausted phenotype, offering a feasible avenue to restore CD8^+^ T cell‐mediated antitumor immunity within cholesterol‐rich tumor microenvironments.

#### 4.2.2 Impact of Altered Membrane Physical Properties on the Immune Synapse

Beyond cholesterol metabolic anomalies, the TME further impairs functional activation by altering the physical properties of the T‐cell membrane, with diminished membrane fluidity serving as a pivotal mechanism. Long‐chain saturated fatty acids (SFAs) accumulating in the interstitial fluid of solid tumors can be uptaken by T cells and integrated into the phospholipid bilayer of their plasma membranes, leading to increased order in lipid packing—that is, enhanced membrane rigidity [113]. This alteration in physical properties directly impacts T‐cell activation efficiency. Efficacious T‐cell activation necessitates the rapid lateral movement and clustering of the TCR and associated co‐stimulatory molecules at the cell‐contact interface to form the immune synapse; however, the rigid cell membrane constrains the lateral diffusion of these signaling proteins, thereby hindering the stable formation of a functional immune synapse. More in‐depth mechanistic studies have revealed that inhibitory receptors, such as PD‐1, due to the characteristics of their transmembrane domains, are more predisposed to enriching within rigid membrane microdomains and forming stable inhibitory signaling complexes, which further antagonize the transduction of activation signals [114]. Consequently, the TME‐induced remodeling of membrane lipid composition is not merely a metabolic side effect; it functions as a putative physical immune regulatory mechanism that, by elevating the activation threshold of T cells, diminishes their responsiveness to low‐affinity tumor antigens.

### 4.3 Lysosomal Dysfunction and Impairments in the Autophagic Process

#### 4.3.1 Lysosomal Acidification Defects

Lysosomes serve as the primary degradative organelles within cells; in cytotoxic T lymphocytes (CTLs), they are further specialized into cytotoxic granules that sequester perforin and granzymes. The activity and stability of these effector proteins are strictly dependent on the acidic environment within the lysosomal lumen [115]. However, the pronounced Warburg effect in solid tumors leads to a massive accumulation of lactate within the TME. Tumor‐derived lactate can be uptaken by T cells via the monocarboxylate transporter 1 (MCT1), inducing cytosolic acidification and thereby disrupting the thermodynamic driving force requisite for maintaining the lysosomal proton gradient [116]. More significantly, the decline in intracellular pH interferes with the proper assembly and function of the vacuolar H^+^‐ATPase (V‐ATPase), leading to an elevation of intralysosomal pH—a phenomenon termed acidification defect. This disruption of pH homeostasis suppresses the cytotoxic function of T cells via two distinct mechanisms [117]. First, it impairs the maturation and pore‐forming capacity of perforin, rendering CTLs capable of contacting target cells but unable to effectively initiate the killing program. Second, proper lysosomal acidification is a prerequisite for its directional transport along the microtubule system toward the immune synapse and its subsequent fusion with the plasma membrane; the loss of the acidic environment thwarts this trafficking process. Consequently, the accumulation of lactate within the TME is likely a contributing factor that impairs the effector functions of T cells at the physicochemical level by disrupting lysosomal pH homeostasis.

#### 4.3.2 Blockade of Autophagic Flux

Autophagy represents a vital pathway for T cells to maintain intracellular homeostasis and achieve material recycling under metabolic stress. During the early stages of T‐cell functional stress, the autophagic pathway is typically activated as an adaptive response to sustain cell survival; however, as cells progress into the terminal Tex state, a distinct impairment in autophagic flux develops. This phenomenon is primarily linked to lysosomal dysfunction [118]. This autophagic stalling separates the resilient Tpex pool from the structurally collapsing terminal subsets. As previously elucidated, the impairment of lysosomal acidification and enzymatic activity prevents the effective fusion of autophagosomes with lysosomes, or results in the failure to degrade cargo post‐fusion. Consequently, the autophagy adaptor protein p62/SQSTM1 and damaged organelles destined for degradation accumulate aberrantly within the cytosol [119]. Research indicates that this blockade of autophagic flux induces significant cellular stress, which not only hinders the capacity for metabolic recycling through degradation but also fosters the accumulation of protein aggregates that may further trigger ROS bursts and compromise genomic stability [107]. Therefore, autophagic dysfunction not only attenuates the adaptive capacity of exhausted T cells within the nutrient‐deprived TME but also potentially propels the transition from functional impairment toward cell death.

#### 4.3.3 Dysregulation of the mTORC1‐TFEB Nutrient‐Sensing Axis

The lysosomal membrane serves not only as an interface for degradative activities but also as a critical hub for nutrient sensing and the regulation of metabolic states. Central to this process is the mechanistic target of rapamycin complex 1 (mTORC1) complex, which localizes to the lysosomal membrane via the Rag GTPase system to monitor amino acid availability [120, 121]. In the TME of solid tumors, the competitive consumption of amino acids by tumor cells leads to impaired amino acid‐sensing mechanisms on the T‐cell lysosomal surface. Specifically, the scarcity of arginine prevents the stable activation of mTORC1 on the lysosomal membrane, thereby suppressing downstream anabolic pathways such as protein synthesis and ribosomal biogenesis [122, 123]. Concurrently, the diminished activity of mTORC1 should theoretically relieve its inhibitory phosphorylation of the transcription factor transcription factor EB (TFEB), promoting its nuclear translocation to activate genes associated with lysosomal biogenesis [124]. However, in Terminal Tex, dephosphorylated TFEB frequently fails to effectively initiate its target gene transcriptional programs, often due to inefficient nuclear import or interference from other regulatory mechanisms. Consequently, the inactivation of mTORC1 coupled with the failure of TFEB‐mediated adaptation creates a cumulative deleterious effect: T cells can neither initiate the anabolism required for proliferation nor enhance lysosomal degradative capacity to cope with stress. This dysregulation of the core nutrient‐sensing axis reflects a significant decline in the ability of exhausted T cells to recalibrate their metabolic status in response to environmental nutrient fluctuations.

### 4.4 Abnormal Inter‐Organelle Interactions

#### 4.4.1 Dysfunction of Mitochondrial‐ER Contact Sites

Mitochondria and the ER do not exist in isolation; they are intricately linked via specialized membrane contact structures known as mitochondria‐associated membranes (MAMs) to facilitate the rapid exchange of signals and materials. In functionally normal effector T cells (Teffs), MAMs serve as critical sites for calcium ion (Ca^2^^+^) transfer, ensuring that Ca^2^^+^ stored in the ER can efficiently enter the mitochondrial matrix through the IP3R‐GRP75‐VDAC complex [125, 126]. This mitochondrial uptake of Ca^2^^+^ is essential for activating key enzymes within the tricarboxylic acid (TCA) cycle, thereby swiftly converting the calcium signals elicited by TCR activation into the metabolic driving force for ATP generation—achieving effective coupling between signal transduction and metabolic activities. However, within the TME, the MAMs structure in Terminal Tex is significantly reduced, leading to profound functional uncoupling. Conversely, Tpex highly depend on intact ER‐mitochondria tethering to coordinate metabolic agility. The mechanism is linked to the aforementioned downregulation of the mitochondrial fusion protein mitofusin‐2 (Mfn2), which also serves as a pivotal protein for maintaining the structural integrity of MAMs. The reduction of Mfn2 increases the distance between the ER and mitochondria, disrupting the microenvironment required for Ca^2^^+^ transfer [127]. This results in two primary functional consequences: first, the lack of sufficient Ca^2^^+^ stimulation in the mitochondria leads to a decline in TCA cycle efficiency, making it difficult to maintain OXPHOS and impairing metabolic flexibility even in the presence of metabolic substrates; second, aberrant fluctuations in cytosolic calcium dynamics may induce abnormal NFAT transcriptional activity, which is predisposed toward initiating the expression of exhaustion‐related genes rather than effector genes. Therefore, the structural and functional disruption of MAMs significantly compromises the metabolic support capacity required for T cells to transform antigen‐recognition signals into sustained effector functions.

#### 4.4.2 Dysfunction of ER‐Lysosome Contact Sites

**FIGURE 4:**
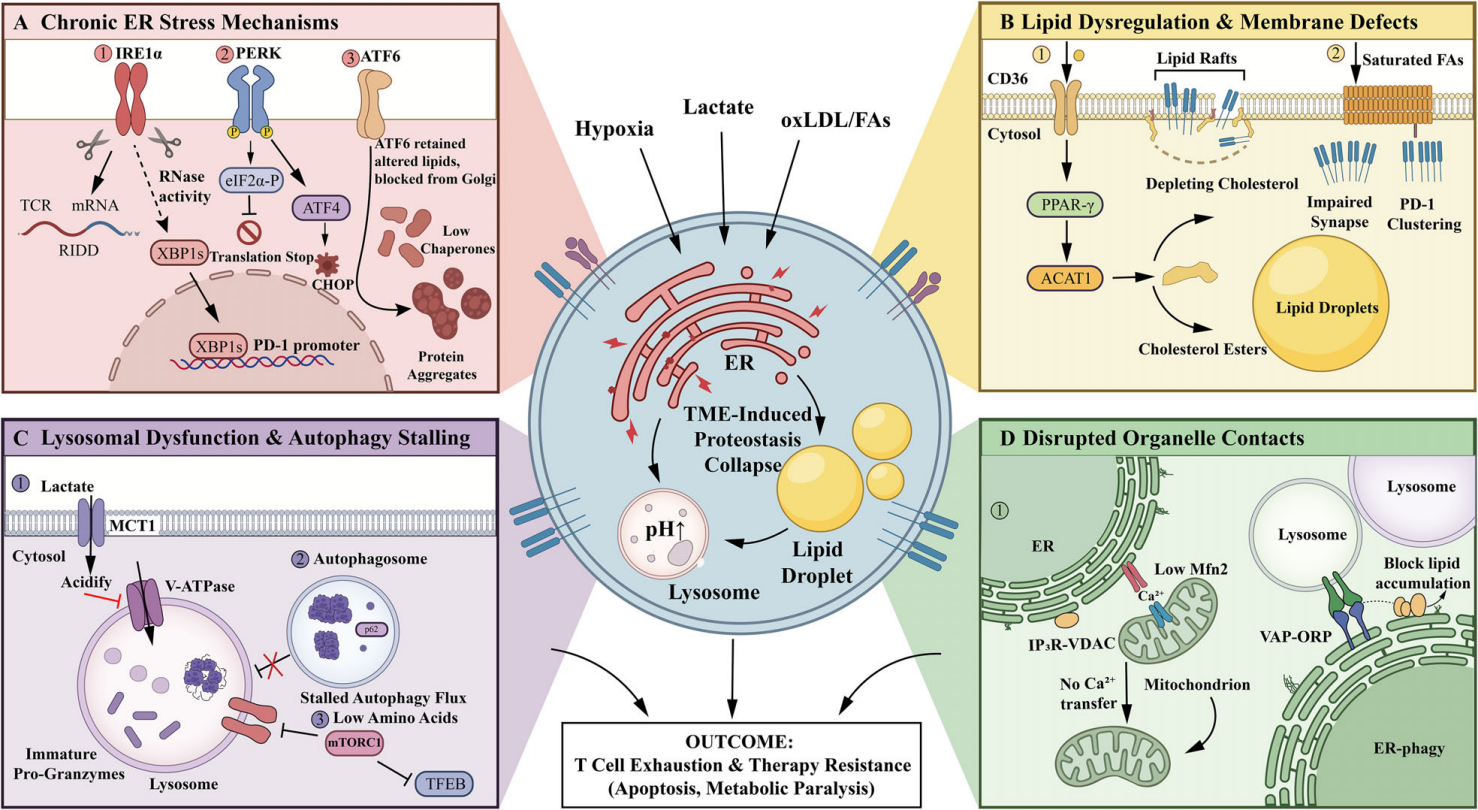
ER stress‐mediated disruption of protein/lipid homeostasis and organelle network communication. [Correction added on 10 June 2026 after online publication: non final version was used and is now replaced with the corrected version.]

Beyond mitochondrial‐ER contacts, the membrane contact structures between the ER and lysosomes also play a significant role in regulating organelle quality control and lipid transport [128]. In functionally normal T cells, when ER stress occurs due to unfolded proteins, ER‐lysosome contact sites can recruit autophagy receptors, such as family with sequence similarity 134 member B (FAM134B), thereby initiating ER‐phagy to eliminate damaged ER fragments and maintain proteostasis [129]. However, within lipid‐enriched TMEs, aberrant lipid accumulation in Terminal Tex interferes with the interactions between key proteins that stabilize these contact sites, such as the vesicle‐associated membrane protein‐associated protein (VAP) and oxysterol‐binding protein‐related protein (ORP) families [130]. The excessive deposition of cholesterol at these contact sites increases membrane rigidity, subsequently hindering the membrane‐contact‐dependent lipid exchange between the ER and lysosomes. This disruption of contact site functionality prevents the effective clearance of damaged ER sequestering misfolded proteins, causing the UPR signaling to persistently skew toward a pro‐apoptotic output. Furthermore, phospholipids synthesized in the ER cannot be efficiently transported to the lysosomal membrane; alterations in the membrane lipid composition affect the stability of V‐ATPase, thereby compromising lysosomal acidification and enzymatic activity. Consequently, the functional loss of ER‐lysosome interactions not only exacerbates proteostasis stress but also impedes the necessary organelle remodeling required for adaptation to stress, leading to a progressive decline in the self‐repair capacity of T cells under the sustained pressure of the TME (Figure 4).

## 5 Nuclear Mechanics and Epigenetic Regulatory Anomalies of Anti‐Tumor T Cells

Within the TME of solid tumors, the aberrant remodeling of the extracellular matrix (ECM) constitutes the primary physical barrier confronting T‐cell infiltration. Compared to physiological lymphoid tissues, cancer‐associated fibroblasts (CAFs) secrete excessive collagen and, through lysyl oxidase (LOX)‐mediated cross‐linking reactions, significantly increase the mechanical stiffness of tumor tissues [131, 132]. This dense physical architecture compels infiltrating T cells to undergo significant nuclear deformation to traverse the narrow interstitial gaps of the ECM. As the stiffest organelle within the cell, the insufficiency of the nucleus's mechanical adaptability not only results in obstructed physical migration but also directly influences the epigenetic state through the conversion of mechanical signals into genomic signals—a process termed mechanotransduction [133]. Therefore, the stiffness of the tumor stroma is not merely a physical parameter; rather, it serves as a critical microenvironmental factor that impairs T‐cell nuclear integrity and chromatin spatial conformation, thereby attenuating the overall efficacy of the anti‐tumor immune response.

### 5.1 Alterations in Nuclear Structure and Mechanosensing

#### 5.1.1 Lamin Remodeling and Nuclear Stiffening

To cope with the high‐stiffness tumor stroma, T cells upregulate the expression of Lamin A/C. While this mechanical adaptation enhances nuclear envelope rigidity to resist external compressive forces, it concurrently and significantly reduces overall nuclear deformability [134, 135]. This increased rigidity, although protective against nuclear envelope rupture, imposes a physical constraint that impedes T cells from traversing narrow interstitial pores, thereby restricting their infiltration into the deep tumor parenchyma. At the pathological level, this markedly hinders T cells from traversing dense matrix gaps, thereby restricting them to the peripheral regions of the tumor parenchyma. This may be one of the reasons for immune exclusion; even if T cells are recruited to the tumor margin, they cannot effectively infiltrate the core area due to insufficient nuclear mechanical adaptability. Furthermore, Lamin A/C anchors heterochromatin to the nuclear periphery through lamina‐associated domains (LADs). Under the continuous mechanical stress of the TME, this anchoring may position critical effector gene loci, including IFNG and GZMB, within transcriptionally repressive perinuclear regions [136]. Consequently, even if a few T cells successfully infiltrate, they may lose their corresponding effector functions due to alterations in chromatin spatial conformation.

#### 5.1.2 Impaired Nuclear Envelope Integrity

When T cells attempt to traverse matrix pores smaller than their nuclear diameter, the mechanical shear forces exerted by the tumor stroma may exceed the endurance limit of the nuclear envelope, resulting in nuclear envelope rupture. The loss of nuclear envelope integrity leads to the disruption of the nucleocytoplasmic barrier; on one hand, nuclear DNA repair factors such as KU70/80 leak into the cytoplasm, while on the other, cytosolic nucleases—prototypically three‐prime repair exonuclease 1 (TREX1)—gain entry into the nucleus to cleave exposed genomic DNA, thereby precipitating extensive DNA double‐strand breaks (DSBs) [137, 138, 139]. Crucially, this mislocalization of TREX1 establishes a profound inter‐organellar feedback loop with the mitochondria. Under physiological conditions, cytosolic TREX1 prevents aberrant immune activation by degrading mislocated cytosolic DNA. However, its massive translocation into the ruptured nucleus leaves the cytoplasm severely depleted of this essential exonuclease. Consequently, when TME‐stressed mitochondria leak mtDNA into the cytosol (as elaborated in Section 3.3), the absence of TREX1‐mediated clearance permits the unrestrained hyperactivation of the cGAS‐STING pathway. This synergistic nuclear‐mitochondrial crosstalk—where physical nuclear damage amplifies mitochondria‐derived inflammatory signaling—accelerates the p53‐p21‐dependent senescence trajectory. Thus, the physical barrier of the tumor stroma confers a specific senescence‐associated mode of immune failure not merely through isolated genomic damage, but by catalyzing a lethal feedback loop between nuclear envelope rupture and mitochondrial signaling cascades [140, 141].

#### 5.1.3 Dysregulation of Nuclear‐Cytoskeletal Mechanical Coupling

Efficient T‐cell migration relies on the mechanical coupling between the nucleoskeleton and the cytoskeleton via the LINC complex (Linker of Nucleoskeleton and Cytoskeleton), which transmits cytoskeletal traction forces to the nucleus to coordinate directional cell movement [142]. However, within the high‐tension TME of solid tumors, persistent mechanical loading may lead to aberrant connections between the key components of the LINC complex [143]. This dysregulation of nuclear‐cytoskeletal coupling impairs the capacity of T cells to sense stromal stiffness gradients, resulting in the loss of directional migration toward the tumor parenchyma, which manifests as random movement or migration arrest within the stroma [144]. This impairment of migratory function prevents T cells from effectively engaging and killing tumor cells, thereby allowing the latter to evade immune surveillance and proliferate within “blind spots” of the immune system.

### 5.2 Epigenetic and Transcriptional Program Solidification

#### 5.2.1 Alterations in Chromatin Accessibility

During acute infections, T cells can rapidly open effector gene loci through chromatin remodeling; however, under chronic antigen stimulation in solid tumors, they exhibit a persistent closed chromatin state. This dysfunctional alteration is primarily associated with the loss of expression of the genome organizer protein special AT‐rich sequence‐binding protein 1 (SATB1). In TILs, microenvironmental signals coupled with continuous TCR signaling suppress the expression of SATB1 [145]. Under physiological conditions, SATB1 maintains the accessibility of regions harboring key effector genes, such as Tcf7 and Bach2, by organizing the higher‐order structure of chromatin. Its absence leads to an aberrant increase in nucleosome density at these loci, forming physical barriers that obstruct transcription initiation and resulting in severely impaired functions, such as cytokine secretion [146]. Therefore, the stabilization of chromatin architecture mediated by SATB1 loss attenuates T‐cell responsiveness to tumor antigens, which may facilitate tumor immune evasion.

#### 5.2.2 Exhaustion‐Related DNA Methylation Modifications

One of the primary factors contributing to the suboptimal clinical response to ICB therapy is the formation of a stable, repressive epigenetic landscape within the genome of the Terminal Tex compartment, particularly within clonotypes possessing high‐affinity TCRs. This process is predominantly driven by the aberrant recruitment and activation of the DNA methyltransferase 3 alpha (Dnmt3a) under conditions of tumor hypoxia and metabolic stress. Dnmt3a executes extensive de novo methylation on the promoter regions of pivotal genes, including those encoding effector and co‐stimulatory molecules; these DNA methylation modifications exhibit profound maintenance stability [147]. The crux of the matter lies in the fact that the stability of these modified marks is governed by a metabolic‐epigenetic axis. For instance, mitochondrial dysfunction and chronic ROS production deplete intracellular α‐ketoglutarate reserves, a metabolite that serves as an indispensable co‐substrate for the TET family of DNA dioxygenases. Once the organellar supply of α‐ketoglutarate becomes limiting, the repressive modifications mediated by Dnmt3a cannot be efficiently erased, thereby locking the cell in a state of exhaustion. Analogously, declining mitochondrial OXPHOS activity reduces the NAD^+^ to NADH ratio, which in turn inhibits the enzymatic activity of NAD^+^‐dependent Sirtuin histone deacetylases. This tight coupling between metabolic and enzymatic processes explains why blockade of the PD‐1 signaling pathway alone often proves insufficient: although such therapy may restore proximal signal transduction, it fails to circumvent the metabolic austerity imposed by organellar dysfunction that initiates and perpetuates the epigenetically locked state. Consequently, even when PD‐1 blockade rescinds the inhibition of the TCR signaling pathway, these methylated gene loci struggle to undergo effective demethylation, and their transcriptional activity remains unrestored. This could offer a potential epigenetic explanation for the mechanism of non‐responsiveness to ICB in certain patients: while PD‐1/PD‐L1 blockade primarily restores signal transduction, it fails to reverse the engrained and more stable DNA methylation imprints. Thus, the combinatorial application of demethylating agents, aimed at erasing these inhibitory marks and reshaping chromatin accessibility, represents a potential strategy to circumvent such resistance mechanisms and reinvigorate the anti‐tumor functionality of T cells.

#### 5.2.3 Clonotypic and Temporal Heterogeneity of the TOX‐Driven Epigenetic Lock

The establishment of an exhaustion‐associated epigenetic lock within the nucleus, centrally orchestrated by the transcription factor TOX, is a dynamic process marked by profound temporal dependence and clonotypic heterogeneity [148]. TOX expression is driven by sustained calcium/NFAT signaling within the TME. In early effector T cells and Tpex cells, chromatin regions encompassing critical stemness genes such as TCF7 remain physically accessible, endowing this subset with essential plasticity for clonal expansion upon ICB intervention. As differentiation advances toward the terminally exhausted phenotype, however, persistently accumulated nuclear TOX recruits HDACs and chromatin remodeling complexes, enforcing irreversible physical anchoring and transcriptional silencing of these key epigenetic loci while directly sustaining high‐level expression of inhibitory receptors including PDCD1 and HAVCR2 [28, 149]. More critically, recent investigations underscore that the rate of nuclear epigenetic consolidation correlates closely with TCR clonotype affinity. High‐affinity T cell clones, upon recognizing cognate tumor antigens, elicit more robust and sustained calcium signaling, precipitating the rapid depletion of their subcellular adaptive reserves. Consequently, high‐affinity clones are among the first to succumb to TOX‐mediated epigenetic silencing and terminal organellar collapse, whereas low‐affinity clones preserve a progenitor‐like transcriptional state for extended durations. This mechanistic framework explicitly demonstrates that nuclear chromatin remodeling and epigenetic exhaustion do not represent a generic, uniform response but rather constitute a time‐dependent pathological trajectory rigorously governed by TCR clonal specificity and lineage status.

**FIGURE 5:**
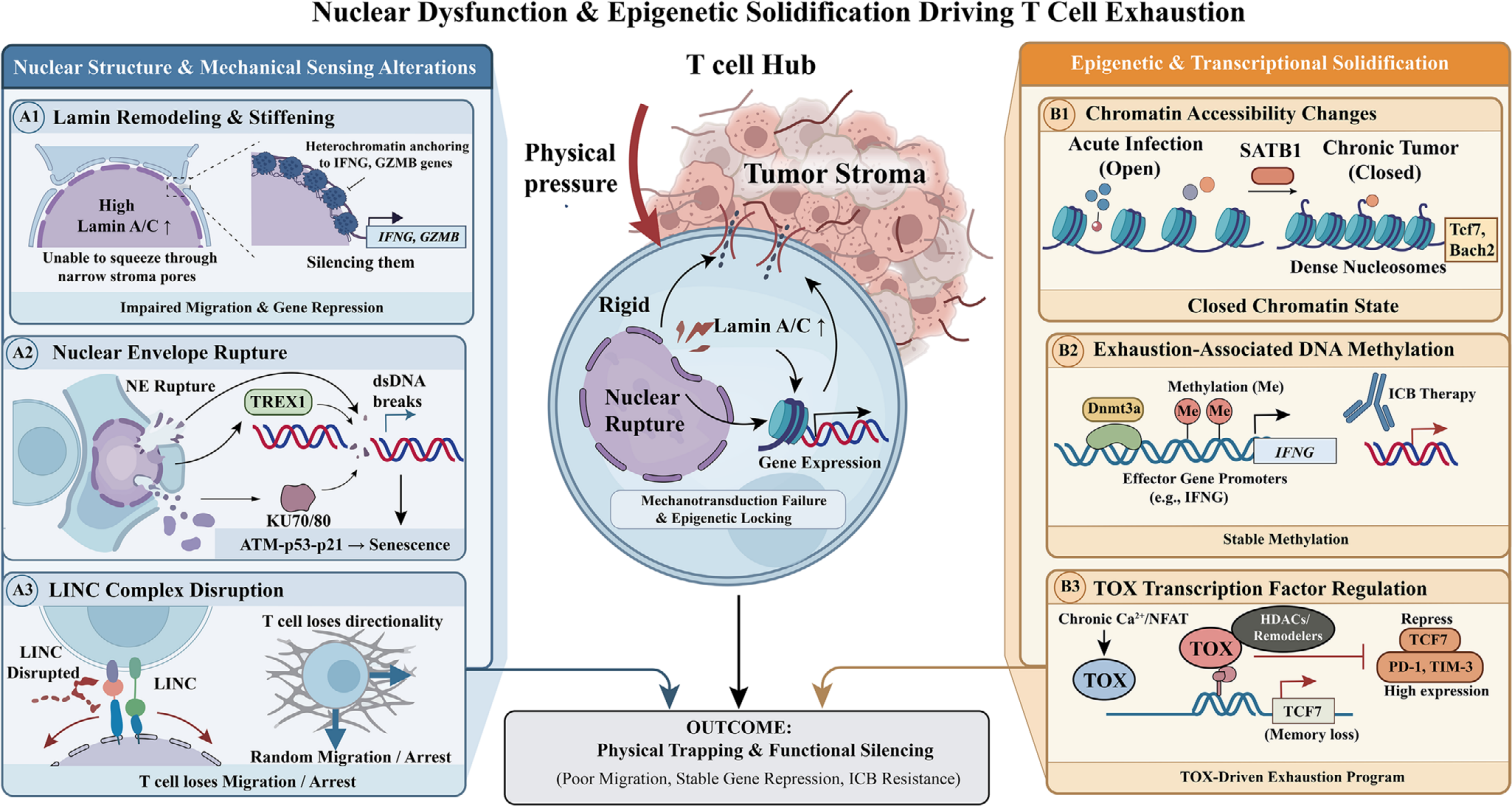
Synergistic exhaustion mechanism driven by mechanophysical stress on the nucleus and deep epigenetic locking.

This hierarchical integrative mechanism fundamentally defines the therapeutic boundaries of subcellular intervention, and its efficacy exhibits pronounced subset specificity. For progenitor exhausted T cells that retain chromatin plasticity, simply rescuing organellar function can effectively reanimate their effector program. For terminally exhausted T cells, however, a strategy focused solely on organellar repair is insufficient. Mechanistically, once the epigenetic lock has been firmly established through stable DNA methylation and TOX‐mediated chromatin sequestration, the underlying transcriptional hardware has undergone obligate and profound reconfiguration. In this state, even physical restoration of mitochondrial or ER homeostasis cannot spontaneously reverse the transcriptional silencing of key effector gene loci. Consequently, to comprehensively dismantle the multidimensional collapse of T cell exhaustion, a synergistic therapeutic approach is theoretically imperative, one that combines organelle‐targeted engineering interventions with epigenetic modifiers to restore metabolic fitness while forcibly unlocking chromatin accessibility (Figure 5).

## 6 Subcellular Dysfunction Characteristics in Different Solid Tumor Microenvironments

Organellar dysfunction is an important common link in T cell exhaustion; however, its specific manifestations exhibit significant heterogeneity among solid tumors of different histological origins. The unique selective pressures inherent to the TME can drive T cells toward distinct pathological trajectories, such as profound exhaustion primarily characterized by mitochondrial metabolic collapse, or a senescence‐like state defined by nuclear damage and genomic instability. Systematically comparing the profiles of T‐cell subcellular dysfunction across different solid tumor types will facilitate the elucidation of the core pathological mechanisms underlying immune resistance, thereby providing a robust rationale for the development of precision therapeutic strategies tailored to specific pathological signatures.

### 6.1 Pancreatic and Breast Cancer

In stroma‐rich malignancies such as pancreatic ductal adenocarcinoma (PDAC) and triple‐negative breast cancer (TNBC), pathological desmoplasia represents a hallmark feature. CAFs excessively secrete and cross‐link type I collagen to construct a high‐stiffness, dense ECM; this physical barrier exerts multi‐dimensional compressive forces on infiltrating T cells [150, 151, 152]. The profound stiffening of the stroma and the elevated solid stress leads to an aberrant elevation in interstitial fluid pressure (IFP), resulting in microvascular hypoperfusion and severe localized hypoxia. Within this extremely hypoxic TME, the OXPHOS of T‐cell mitochondria is profoundly compromised, leading to a precipitous decline in ATP generation. This bioenergetic collapse directly impairs the capacity of T cells to drive actin cytoskeletal rearrangement, culminating in a loss of locomotor function and the subsequent physical sequestration of these cells from the tumor parenchyma. This mechanistic cascade may contribute to the subcellular basis of the clinically observed immune‐excluded phenotype.

For the minority of T cells capable of infiltrating high‐tension matrices, their nuclei endure extreme physical compression. To traverse dense interstitial pores, the nucleus undergoes extreme deformation, often accompanied by disruption of nuclear envelope integrity and activation of the DNA damage response. Studies in breast cancer models have demonstrated that such persistent mechanical insults can activate the ATR‐Chk1 signaling pathway, driving T cells into a specific senescence‐like state. Crucially, distinct from the canonical senescence‐associated secretory phenotype (SASP) conventionally exhibited by stromal or tumor cells, these T cells subjected to chronic mechanical loading develop a highly specific “T cell‐restricted senescence secretory phenotype” (T‐SASP). Rather than secreting classical pro‐inflammatory mediators, this T‐SASP is uniquely enriched in immunosuppressive and profibrotic factors, such as TGF‐β and osteopontin [153]. Notably, these specific T‐SASP factors can reciprocally activate cancer‐associated fibroblasts, further promoting pathological matrix stiffening and establishing a mechanically suppressive feedback loop.

For the minority of T cells that successfully breach the physical barrier of the dense stroma, their lysosomes and ER endure extreme environmental stress. In TNBC, highly glycolytic tumor microregions generate substantial lactate. The dense stroma impedes metabolite clearance, leading to localized accumulation of extracellular lactate to toxic levels. This profoundly acidic milieu irreversibly cripples lysosomal V‐ATPase activity, blocking autophagic flux and depriving T cells of their macromolecular recycling machinery, ultimately culminating in severe metabolic insufficiency [117]. Within the PDAC microenvironment, conversely, profound microvascular hypoperfusion engenders global scarcity of glucose and amino acids. Confronted with nutrient deprivation, T cells are compelled to upregulate CD36 and shift toward reliance on exogenous lipid uptake. Yet the oxidative TME transforms this metabolic adaptation into a trap: excessively internalized lipids overload the ER with oxLDL, triggering lipid peroxidation and the accumulation of misfolded proteins. The terminal UPR IRE1α/CHOP axis becomes persistently activated, driving T cells toward proteotoxic dysfunction and activation‐induced apoptosis [106].

Therefore, immune attrition in these malignancies does not stem from a singular mechanism but rather from the synergistic convergence of sustained mechanical burden, bioenergetic collapse, and localized proteotoxic/metabolic toxicity. Pharmacological agents targeting stromal remodeling can alleviate this composite solid stress. When employed in conjunction with immune checkpoint blockade, such strategies may facilitate the restoration of subcellular functional homeostasis in T cells, ultimately overcoming immune exclusion [154].

### 6.2 Hepatocellular Carcinoma and Glioma

Characterized by a distinct lipid metabolism dysregulation, hepatocellular carcinoma and glioblastoma, unlike stroma‐rich tumors, create a microenvironment where such metabolic disturbances are central to inducing subcellular functional impairment in T cells. In hepatocellular carcinoma, the tumor interstitial fluid is enriched with oxidized low‐density lipoprotein and polyunsaturated fatty acids. Infiltrating CD8^+^ T cells uptake excessive lipids via high expression of the fatty acid transporter CD36, subjecting their mitochondria to severe lipid peroxidation stress [80, 155]. Under physiological conditions, the mitochondrial antioxidant enzyme GPX4 effectively clears lipid peroxides; however, within the hepatocellular carcinoma microenvironment, persistent reactive oxygen species generation coupled with insufficient cystine uptake leads to the functional exhaustion of GPX4 [156, 157]. Failure of this antioxidant defense system leads to irreversible oxidative damage to the mitochondrial membrane, ultimately inducing ferroptosis in T cells. This activation‐induced cell death physically depletes the intratumoral T cell pool, representing a key terminal trajectory of immune failure. Ferroptosis not only exacerbates immunosuppression but may also promote macrophage polarization toward a pro‐tumor phenotype by releasing damage‐associated molecular patterns (DAMPs), thereby establishing a metabolically toxic immunosuppressive microenvironment.

Furthermore, the lipid‐enriched microenvironment of hepatocellular carcinoma directly induces morphological and structural abnormalities in T cell mitochondria. Electron microscopy reveals that mitochondria within infiltrating T cells frequently appear swollen. This phenomenon is attributed to the insertion of excessive intracellular free fatty acids and lipid peroxides into the outer mitochondrial membrane, altering its biophysical properties. These morphologically aberrant mitochondria exhibit a sparse cristae structure and reduced matrix density, and are often accompanied by abnormal opening of the mitochondrial permeability transition pore, leading to severe impairment of their oxidative phosphorylation function [158, 159]. Within the hepatocellular carcinoma microenvironment, the clearance of damaged mitochondria via the PINK1‐Parkin pathway is also frequently suppressed, preventing their timely removal by mitophagy. This allows them to persistently generate reactive oxygen species and leak mitochondrial DNA, thereby exacerbating cellular stress and functional decline [160].

In glioblastoma, T cell dysfunction is primarily attributed to membrane dysfunction arising from cholesterol metabolic reprogramming. Brain tissue is inherently lipid‐rich, and glioma cells, driven by aberrantly activated sterol regulatory element‐binding protein signaling, exacerbate the competitive consumption of cholesterol within the microenvironment [161]. To adapt to this milieu, infiltrating T cells activate ER stress, which impairs cholesterol synthesis; concurrently, the tumor microenvironment downregulates SREBP2 signaling in these cells, further diminishing the availability of free cholesterol in the plasma membrane [162]. Cholesterol is indispensable for maintaining the structural integrity of membrane lipid rafts and facilitating the assembly of signal transduction complexes, and its depletion directly compromises the transmembrane signaling efficiency of the T cell receptor. Consequently, in glioblastoma, T cell dysfunction predominantly manifests as a signaling defect stemming from altered membrane composition. Even upon direct contact with tumor cells, these T cells are unable to effectively initiate cytolytic programs.

However, the microenvironment exhibits substantial variation across distinct spatial regions. Within the HCC microenvironment, zones of severe hypoxia frequently overlap with lipid‐rich areas. In hypoxic cores, T cells internalize copious lipids via CD36, yet these lipids cannot be efficiently oxidized by mitochondria. Unmetabolized lipids aberrantly accumulate within the ER and lysosomes. Profound lipotoxicity triggers unmitigated ER stress, hyperactivating the IRE1α/CHOP apoptotic axis. Concurrently, lysosomal acidification is impaired due to congestion with lipid droplets, and LAL activity is suppressed. Autophagic flux is consequently completely obstructed, depriving T cells of essential intracellular recycling machinery [96].

The microenvironment of glioblastoma is similarly highly heterogeneous. As infiltrating T cells migrate from the cholesterol‐deprived invasive margin toward the necrotic tumor core, they are simultaneously exposed to extreme hypoxia and elevated oxidative stress. Within these spatial niches, T cells not only manifest membrane signaling defects but also undergo severe mitochondrial bioenergetic collapse. Hypoxia directly suppresses OXPHOS, while heightened ROS provoke pronounced mitochondrial fragmentation and loss of membrane potential. A state of terminal metabolic paralysis is thereby established, compounding the cholesterol‐driven signaling failure [59]. Thus, within these specific TME niches, immune evasion results from the synergistic interplay of lipid‐driven membrane disruption, unrelenting organellar lipotoxicity, and local hypoxic bioenergetic failure.

### 6.3 Melanoma and Renal Cell Carcinoma

While melanoma and clear cell renal cell carcinoma (ccRCC) are conventionally grouped based on their high glycolytic flux [163, 164], their clinical response rates to immune checkpoint blockade (ICB) exhibit significant divergence, suggesting distinct underlying subcellular fingerprints. In both malignancies, the Warburg effect results in the massive accumulation of lactate within the TME, establishing a chemically suppressive milieu characterized by acidosis. High extracellular lactate concentrations disrupt the lactate efflux gradient that T cells depend on via monocarboxylate transporters (MCTs), leading to the pathological intracellular accumulation of lactate and inducing cytosolic acidification. This directly inhibits the activity of phosphofructokinase (PFK), a rate‐limiting enzyme of glycolysis, thereby obstructing the carbon flux required for the proliferation of Teffs. More significantly, the shifted equilibrium of the lactate dehydrogenase (LDH) reaction leads to a decreased intracellular NAD+/NADH ratio, triggering reductive stress [165]. This loss of redox homeostasis suppresses the activity of the NAD+‐dependent deacetylases, Sirtuins, resulting in aberrantly elevated acetylation levels of histones and non‐histone proteins; this, in turn, epigenetically silences the transcription of effector genes such as IFNG, providing a mechanistic explanation for how acidic microenvironments induce T‐cell dysfunction through metabolic‐epigenetic coupling [166]. Furthermore, these tumor cells competitively sequester glucose from the microenvironment via the high expression of glucose transporters (GLUTs), causing severe localized glucose deprivation. Under these glucose‐restricted conditions, the synthesis of glycolytic intermediates in T cells is hindered, disrupting the calcium signaling oscillations mediated by the sarco/ER Ca2+‐ATPase (SERCA) pump and leading to insufficient activation of NFAT [167]. Simultaneously, energy‐sensing mechanisms activate AMP‐activated protein kinase (AMPK), which subsequently inhibits the mTORC1 signaling pathway. The inactivation of mTORC1 directly blocks downstream translation initiation and ribosomal biogenesis, thereby suppressing the anabolism and clonal expansion of T cells [64].

ccRCC and melanoma also exhibit significant glutamine dependence, upregulating glutaminase (GLS) to consume vast quantities of glutamine from the TME to sustain the TCA cycle [168]. Glutamine serves as a precursor for the synthesis of the critical antioxidant glutathione (GSH); its deprivation weakens the antioxidant defense capacity of T cells, inducing secondary oxidative stress and mitochondrial damage. Furthermore, glutamine is an indispensable signaling molecule for maintaining the protein stability of the transcription factor cellular myelocytomatosis oncogene (c‐Myc). Under conditions of glutamine deprivation, T cells encounter impairments in c‐Myc‐dependent metabolic reprogramming, rendering them unable to initiate metabolic adaptation programs associated with activation [169].

However, the subcellular injury network in melanoma and ccRCC is far more complex than simple nutrient competition. Mechanistically, in ccRCC, pervasive loss of the VHL tumor suppressor results in constitutive stabilization of HIF‐2α signaling. Beyond driving the aforementioned glycolysis, this signaling axis uniquely reprograms lipid metabolism, leading to substantial accumulation of cholesterol and neutral lipids within the TME. Consequently, tumor‐infiltrating CD8^+^ T cells in ccRCC confront a distinct lipid stress. This clear cell phenotype microenvironment not only imposes a pseudohypoxic state upon infiltrating T cells, but its accumulated lipid debris also induces lysosomal lipid deposition in T cells. Such lipid droplet engorgement suppresses LAL activity, crippling autophagy‐mediated organelle renewal and leaving damaged mitochondria uncleared, thereby perpetually generating endogenous ROS [117]. Moreover, this extreme composite stress of lipid overload and hypoxia provokes marked disruption of MAM architecture within T cells. The rupture of inter‐organelle communication severely perturbs precise calcium shuttling, ultimately precipitating mitochondrial metabolic paralysis and loss of effector function [170].

This contrasts with the metabolic fingerprint of melanoma. Within the melanoma microenvironment, beyond the pervasive acidosis mediated by high lactate, the efflux of metabolic intermediates from the melanin synthesis pathway constitutes a unique biochemical stress. These moieties impose severe secretory stress upon the Golgi apparatus of T cells, the fragmentation of which not only suppresses effector protein maturation but also impairs polarized cytokine trafficking. Concurrently, the highly acidic lactate microenvironment specifically induces lysosomal membrane permeabilization in T cells, leading to cathepsin leakage into the cytosol and direct cleavage of key signal transduction proteins. Such sublethal structural compromise, in conjunction with epigenetic exhaustion, may provide a mechanistic basis for resistance to immunotherapy [171].

In summary, although both tumor types share glycolysis as a metabolic hallmark, their patterns of organellar stress diverge markedly. In ccRCC, the stress landscape is dominated by VHL/HIF axis‐driven lipotoxicity and hypoxic stress, whereas in melanoma it is characterized by lysosomal acidification impairment and Golgi stress induced by extreme lactate accumulation. These two distinct subcellular stress signatures dictate divergent trajectories of T cell exhaustion. Such mechanistic divergence at the subcellular level provides a critical biological foundation for interpreting the disparate therapeutic sensitivities these malignancies exhibit toward immune checkpoint blockade [167].

### 6.4 NSCLC

In the TME of NSCLC, vigorous oxidative metabolism coupled with intermittent hypoxia results in persistently high levels of ROS; this oxidative stress poses a significant threat to the mitochondrial genome of infiltrating T cells [172]. Because mtDNA lacks histone protection and possesses limited repair capacity, excessive superoxide anions in the NSCLC TME readily attack T‐cell mtDNA directly, leading to oxidative damage and the accumulation of mutations within its coding regions [173]. Research indicates that T cells infiltrating NSCLC frequently exhibit functional defects in ETC complexes I/III, which are direct consequences of mtDNA damage [172]. The impairment of the respiratory chain further exacerbates electron leakage, thereby generating even more ROS and establishing a self‐reinforcing cycle. The resultant mitochondrial membrane potential (MMP) depolarization and cristae disorganization not only reduce ATP synthesis but may also trigger the sublethal release of cytochrome c. The latter can activate caspase‐3, which subsequently cleaves and inactivates key nuclear transcription factors such as NFAT and nuclear factor kappa B (NF‐κB), thereby causing T cells to lose critical signal transduction and effector gene activation capabilities. This manifests as a state of functional suppression dominated by oxidative stress [49].

However, the microenvironment of NSCLC is not uniformly hyperoxidized; its profound spatial heterogeneity exposes infiltrating T cells to multidimensional subcellular pressures across distinct regions. In the profoundly ischemic core distal to functional vasculature, the dual deprivation of oxygen and nutrients not only exacerbates mitochondrial collapse but also directly disrupts oxidative folding homeostasis within the ER. Aberrant accumulation of unfolded proteins within the ER lumen triggers intense ER stress, and the persistently activated terminal UPR further drives T cells toward proteotoxic dysfunction [96]. Concurrently, in regions of extreme ROS abundance, excessive oxidative stress not only assaults mtDNA but also directly induces lipid peroxidation of the lysosomal membrane, precipitating LMP. This not only completely cripples autophagy‐mediated organellar quality control and metabolite recycling but also results in cathepsin leakage into the cytosol. The leaked cathepsins, in concert with the caspase‐3 cascade released from the aforementioned mitochondria, further cleave key signaling molecules and accelerate the functional decline and noncanonical death pathways of T cells [174].

Therefore, within the intricate spatial microenvironment of NSCLC, loss of immune competence is not attributable to a singular mechanism but rather represents a composite outcome interwoven with mitochondrial genome collapse, ER proteotoxic stress, and lysosomal structural disruption. This multi‐organellar cascade failure comprehensively deprives T cells of signal transduction and survival capacity, underscoring the comprehensive contribution of intratumoral spatial heterogeneity to immune evasion.

### 6.5 Colorectal Cancer

Within the unique intestinal microenvironment of colorectal cancer (CRC), the interaction between tumor cells and the gut microbiota significantly alters the composition of local metabolites, characterized by an aberrant ratio of short‐chain fatty acids (SCFAs) and the massive accumulation of succinate. This metabolic milieu directly impacts mitochondrial dynamics by interfering with the TCA cycle, leading to dysfunctional alterations primarily manifested as excessive mitochondrial fission [175, 176]. High concentrations of succinate inhibit prolyl hydroxylase (PHD) activity, resulting in the stabilization and pseudohypoxic activation of HIF‐1α, which subsequently upregulates the transcription of the fission protein Drp1 and suppresses the expression of the fusion proteins Mfn1/2 [177]. This dynamic imbalance causes the originally contiguous mitochondrial network within T cells to fragment into discontinuous segments, markedly reducing the effective surface area of mitochondrial cristae and the assembly efficiency of respiratory chain supercomplexes. Consequently, infiltrating T cells struggle to maintain efficacious OXPHOS to support sustained immune synapse functionality, thereby offering a potential subcellular explanation for why microsatellite stable (MSS) CRC frequently exhibits a “cold tumor” phenotype with poor responsiveness to immunotherapy [178].

Furthermore, the CRC microenvironment exhibits a highly stratified spatial metabolic heterogeneity. Local spatial gradients and regionally distributed bacterial biofilms intertwine to collectively dictate the subcellular stress modalities encountered by infiltrating T cells. Within the deeply invasive tumor core, succinate‐driven mitochondrial fragmentation is further exacerbated by RET at mitochondrial complex I. Excessive oxidation of high‐concentration succinate drives a catastrophic burst of mitochondrial ROS, and this uncontrolled oxidative outburst not only induces secondary mtDNA damage but also inflicts severe lipid peroxidation upon adjacent ER membranes, thereby triggering unmitigated ER stress and proteotoxic dysfunction [179].

Conversely, at the mucosal‐invasive interface colonized by polymicrobial biofilms, infiltrating T cells are enveloped within a distinct biochemical milieu dominated by dysregulated bacterial metabolites. Although physiological levels of SCFAs contribute to the maintenance of intestinal homeostasis, pathologically elevated concentrations of SCFAs at the biofilm periphery act as potent HDAC inhibitors, enforcing aberrant epigenetic hyperacetylation in T cells that stably silences effector programs and establishes a microbiome‐driven epigenetic lock [180]. Simultaneously, excessive local accumulation of highly hydrophobic secondary bile acids serves as a potent biological detergent, and these aberrant metabolites physically disrupt the lipid bilayers of the endolysosomal network within T cells, inducing LMP. The resultant leakage of lysosomal hydrolases completely derails autophagic flux and accelerates the comprehensive structural and functional decline of T cells [181].

Therefore, within the unique intestinal microenvironment of CRC, immune exclusion is not attributable to a singular mitochondrial defect but rather represents a composite process deeply interwoven with succinate‐driven mitochondrial fragmentation, RET‐induced ER proteotoxicity, and biofilm‐derived epigenetic and lysosomal physical disruption.

### 6.6 Hematologic Malignancies

Within the bone marrow microenvironment (BMM) of leukemia and multiple myeloma (MM), malignant cells can interfere with T‐cell cytoskeletal dynamics and organelle trafficking through direct contact, leading to functional impairment. Compared to solid tumors, T‐cell dysfunction in hematologic malignancies is prominently characterized by defects in organelle polarization during the formation of the immune synapse (IS) [182]. Under physiological conditions, the killing efficacy of CTLs depends on the rapid, directional translocation of the microtubule‐organizing center (MTOC) toward the IS, which subsequently directs critical organelles, such as lysosomes and mitochondria, to aggregate at the contact interface. However, in models such as chronic lymphocytic leukemia (CLL), tumor cells can suppress the activity of the Rho GTPase signaling pathway in T cells, thereby hindering essential actin cytoskeletal remodeling [183]. This defect triggers a cascade of organelle‐level anomalies: mitochondria fail to be effectively recruited to the sub‐synaptic region, resulting in an inadequate local ATP supply, which in turn compromises calcium pump function and may lead to the disruption of synaptic Ca^2^^+^ homeostasis [184]. Concurrently, perforin‐loaded lysosomes cannot be accurately transported along the microtubule system to fuse with the plasma membrane. This impairment of subcellular trafficking and positioning confines the interaction between the T cell and its target to the recognition phase, precluding the formation of a mature, lytic IS capable of effective killing [185].

Beyond direct cell contact inhibition and organelle polarization defects, the bone marrow microenvironment exhibits highly specialized spatial regional heterogeneity. Lipid‐rich niches dominated by bone marrow adipocytes constitute an additional stringent subcellular metabolic barrier. In multiple myeloma and certain leukemias, malignant cells remodel bone marrow adipocytes, compelling them to release abundant free fatty acids into the microenvironment. Infiltrating T cells within these regions are forced into excessive lipid uptake, inevitably precipitating severe ER lipotoxicity. Sustained lipid overload not only disrupts oxidative folding homeostasis within the ER, triggering a robust unfolded protein response, but also leads to aberrant lysosomal lipid deposition. Such lipid droplet engorgement directly cripples lysosomal lipophagic degradation, rendering damaged organelles ineffectively cleared and ultimately driving T cells toward dual metabolic and structural failure [186].

Simultaneously, within the profoundly hypoxic regions immediately adjacent to the endosteal surface, T cells confront a fundamentally distinct biochemical stress. This niche is characterized not only by extremely low oxygen tension but also by marked enrichment of adenosine generated via the concerted ectoenzymatic activity of CD39 and CD73. Elevated adenosine concentrations engage the A2A receptor on the T cell surface, precipitating an intracellular surge in cAMP and potent activation of PKA signaling. Aberrant PKA activation not only directly phosphorylates and inhibits core metabolic enzymes within mitochondria but also transmits potent suppressive signals to the nucleus, culminating in irreversible epigenetic silencing of chromatin regions associated with effector function [187].

In summary, T cell dysfunction in hematological malignancies arises not only from cytoskeletal and organellar spatial positioning defects during immunological synapse assembly but is further compounded by ER‐lysosomal paralysis induced by lipid overload within specific bone marrow niches, as well as mitochondrial‐nuclear signaling blockade driven by the hypoxia‐adenosine axis. Multidimensional subcellular stress is thus recognized as a critical synergistic contributor to immune evasion within the bone marrow microenvironment.

### 6.7 Prostate Cancer

In the TME of prostate cancer (PCa), the profound depletion of tryptophan coupled with a persistent viral antigen load subjects infiltrating T cells to a state of chronic integrated stress response (ISR) [188]. The scarcity of amino acids such as tryptophan activates general control nonderepressible 2 (GCN2) kinase, leading to the sustained phosphorylation of its downstream target, eIF2α, which globally inhibits cap‐dependent protein translation. To cope with the resulting accumulation of unfolded proteins, the ER of T cells undergoes compensatory structural expansion, manifested as the swelling and disorganized arrangement of the ER lumen—morphological alterations that signify a severe breakdown of ER proteostasis [189]. During this phase, the persistently high expression of transcription factors such as ATF4 and CHOP shifts their function from adaptive repair to the suppression of critical effector transcription factors, including T‐bet and Eomes [102]. Concurrently, ER stress triggers the release of excessive calcium ions through IP3 receptors (IP3Rs) into the mitochondria, disrupting the normal functional coupling at MAMs [190, 191].

Beyond extensive tryptophan consumption, the prostate cancer microenvironment and similarly profoundly immunosuppressive milieus exhibit marked spatial heterogeneity in amino acid metabolism. In advanced prostate cancer, particularly within densely fibrotic regions or the bone metastatic microenvironment, substantial spatial infiltration of MDSCs is evident. These focally aggregated MDSCs secrete exceptionally high levels of ARG1, creating microregional niches of profound arginine deprivation [53]. For T cells traversing these arginine‐depleted zones, the subcellular insult extends beyond ER stress and more critically targets the endolysosomal network.

Under physiological conditions, arginine serves as an obligate metabolic signal for the SLC38A9 nutrient‐sensing complex, enabling its anchorage on the lysosomal surface and subsequent activation of mTORC1 [192]. Under conditions of absolute microenvironmental arginine deprivation, mTORC1 undergoes physical dissociation from the lysosomal membrane, completely crippling its kinase activity. This lysosome‐centered signaling collapse not only abrogates the anabolic pathways requisite for T cell proliferation but also unleashes a dysregulated and destructive state of excessive autophagy. In the absence of compensatory organelle biogenesis, this persistent and futile autophagic degradation severely erodes mitochondrial mass and compromises functional integrity, propelling T cells toward irreversible metabolic attrition and structural atrophy [193].

**FIGURE 6:**
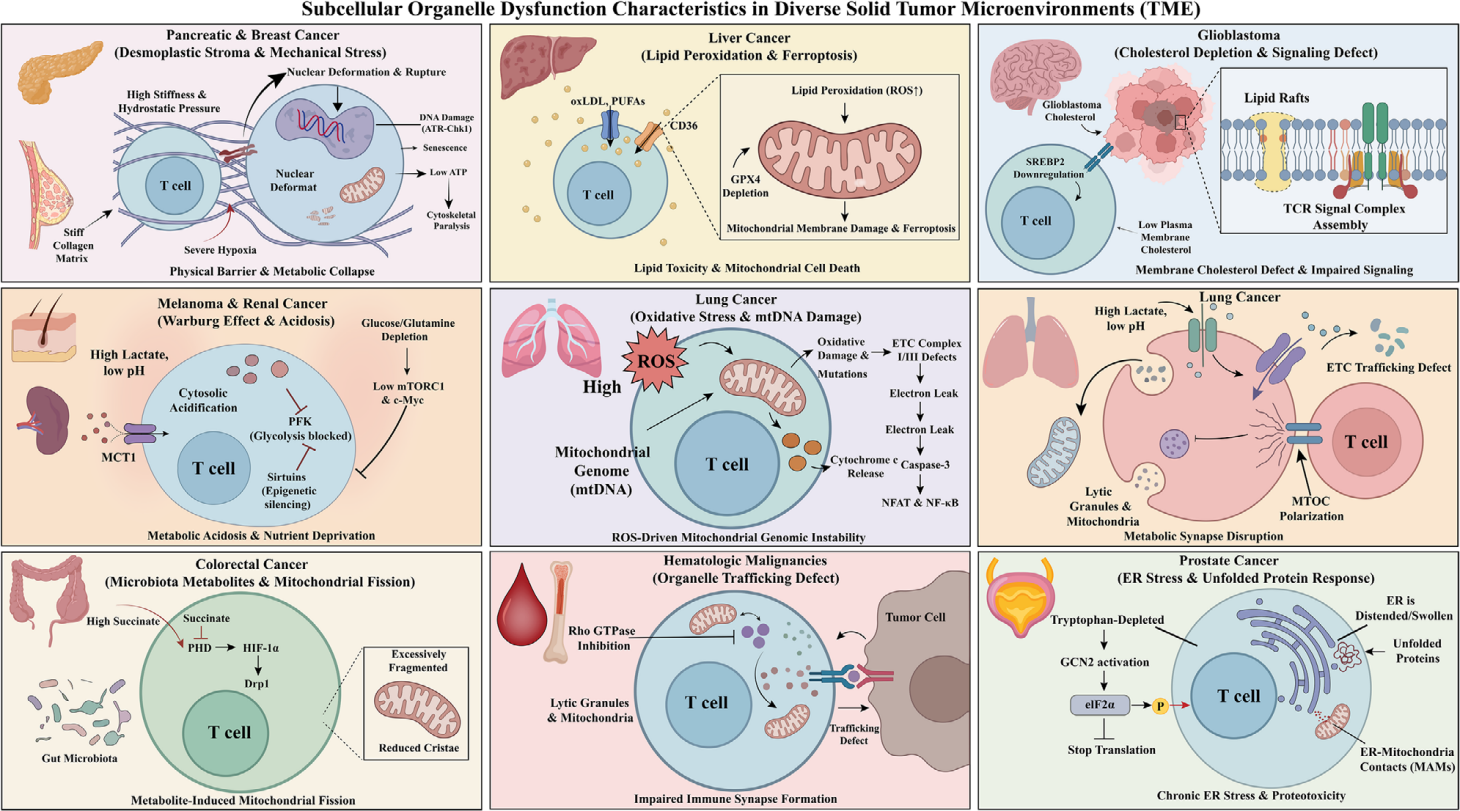
Subcellular pathological heterogeneity of T cells driven by the physicochemical specificity of the pan‐cancer microenvironment.

Therefore, within these microenvironments characterized by specific amino acid deprivation, immune attrition represents a multidimensional composite outcome encompassing GCN2‐mediated ER proteostatic collapse, dysregulated MAM calcium shuttling, and MDSC‐driven lysosomal mTORC1 signaling paralysis. This mechanistic framework elucidates how discrete nutrient scarcities within the TME can initiate a cascading, synergistic failure across multiple organelles (Figure 6).

## 7 Subcellular Functional Discrepancies among T‐Cell Subsets Within the TME

T‐cell exhaustion exhibits significant heterogeneity across various lineages, originating from their distinct effector functions, metabolic requirements, and adaptive capacities within the TME. Due to their elevated metabolic demands, the mitochondrial networks of CD8^+^ Teffs are particularly susceptible to functional and structural dysregulation in nutrient‐deprived TMEs; conversely, Tregs acquire enhanced environmental tolerance through specialized organelle adaptation mechanisms. This lineage‐specific variation in subcellular adaptive capacity strongly influences the functional equilibrium between effector and suppressive immune populations within the TME.

### 7.1 CD8^+^ T Cells

As the primary effectors of anti‐tumor immunity, CD8^+^ T cells are profoundly dependent on an intact cytoskeleton and a functional mitochondrial network. In the TME, their prominent subcellular dysfunction first manifests within the lysosomal secretory granule system. To achieve efficacious killing, the centrosome must guide perforin‐loaded lysosomes along microtubules to undergo directional migration toward the IS. However, TME‐induced dysregulation of cholesterol metabolism alters the lipid raft properties of the CD8^+^ T‐cell membrane, interfering with centrosome anchoring at the plasma membrane and subsequently resulting in degranulation failure [194, 195]. Simultaneously, the high‐intensity cytoskeletal rearrangements required for CD8^+^ T‐cell function necessitate significant ATP support, rendering these cells particularly susceptible to mitochondrial dysfunction. Hypoxia combined with persistent antigenic stimulation promotes Drp1‐mediated mitochondrial hyper‐fission, leading to intracellular mitochondrial fragmentation [196]. This morphological alteration not only reduces effective ATP generation but also facilitates the excessive production of ROS. The downstream consequence is that even when signaling pathways such as NFAT are activated, they fail to effectively drive the transcription of effector genes, thereby establishing a state of terminal dysfunction characterized by severe bioenergetic collapse and physical energy constraints.

### 7.2 CD4^+^ T Cells

Unlike CD8^+^ T cells, the subcellular stressors encountered by CD4^+^ T cells within the TME are more likely to result in the dysregulation of their functional lineage stability. The differentiation trajectory of CD4^+^ T cells is finely tuned by mitochondrial redox status. Within the TME, excessive ROS generated by mitochondrial complex III not only inflict oxidative damage but also serve as aberrant signaling molecules that interfere with the core transcriptional regulatory networks [197]. Research indicates that elevated levels of mitochondrial ROS suppress the mTORC1 signaling pathway, leading to the downregulation of T‐bet, the master transcription factor of T helper 1 (Th1) cells, while simultaneously creating conditions permissive for the expression of regulatory‐associated factors such as forkhead box P3 (FoxP3) [198]. This ROS‐driven lineage imbalance prompts a phenotypic shift in Th1 cells—which should ideally exert auxiliary anti‐tumor functions—into a regulatory‐like state characterized by the secretion of inhibitory cytokines such as interleukin‐10 (IL‐10) [199]. Furthermore, lysosomal dysfunction in CD4^+^ T cells may compromise their antigen presentation capacity mediated by major histocompatibility complex class II (MHC II) molecules, thereby indirectly weakening their help for CD8^+^ T‐cell activation [200].

### 7.3 Regulatory T Cells

**FIGURE 7:**
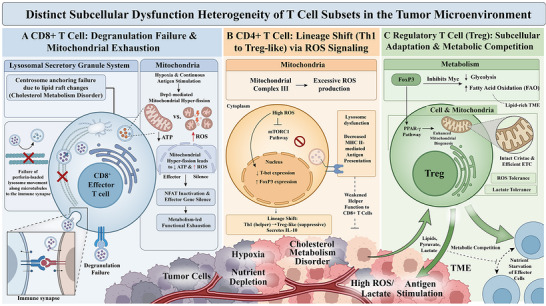
Subcellular functional heterogeneity and metabolic competition among key t cell subsets (CD8^+^, CD4^+^, Treg) in the tumor microenvironment.

Unlike the generalized functional impairment observed in Teffs within the TME, Tregs frequently exhibit robust subcellular adaptability, a survival advantage rooted in their unique metabolic programs. The high expression of the transcription factor FoxP3 in Tregs suppresses Myc, thereby reducing glycolytic activity and biasing their metabolism toward FAO for energy supply [201]. In lipid‐enriched TMEs, this metabolic paradigm allows Tregs to utilize abundant lipid resources more efficiently. The mitochondrial networks in Tregs are not only functionally maintained but may also undergo enhanced mitochondrial biogenesis via pathways such as PPAR‐γ, thereby preserving relatively intact cristae architecture and ETC efficiency. This endows Tregs with superior tolerance to metabolic stressors such as ROS and lactate [202, 203]. More significantly, by virtue of their active OXPHOS, Tregs can efficiently sequester metabolic substrates like fatty acids and pyruvate from the microenvironment [204]. This metabolic competition further exacerbates the nutrient scarcity faced by Teffs. Consequently, by maintaining superior subcellular functional homeostasis, Tregs not only ensure their own survival but also establish a dominant metabolic competitive advantage within the TME (Figure 7).

## 8 Organelle Therapeutic Targets Within the TME

### 8.1 MCJ

The efficiency of the mitochondrial ETC is a critical determinant of the ATP generation rate and effector functionality of T cells. Methylation‐controlled J protein (MCJ, also known as DnaJC15), an endogenous negative regulator localized to the inner mitochondrial membrane (IMM), inhibits the formation of respiratory chain supercomplexes by interfering with the assembly of complex I with complexes III/IV, thereby limiting maximal respiratory capacity. Research by Champagne et al. confirmed that MCJ acts as a “metabolic brake”; its genetic deletion significantly promotes supercomplex assembly, enhancing the OXPHOS capacity and MMP of CD8^+^ T cells, which in turn increases IFN‐∖gamma secretion and the efficiency of viral clearance [205]. Subsequent investigations in melanoma models further validated this strategy, demonstrating that MCJ‐deficient T cells exhibit improved resilience to metabolic stressors within the TME and manifest potent cytotoxicity [206]. Consequently, targeted silencing of MCJ via siRNA or small‐molecule inhibitors offers an effective avenue to reverse metabolic suppression and unleash the bioenergetic potential of anti‐tumor T cells.

### 8.2 PGC‐1α

Within the TME of solid tumors, persistent TCR stimulation and hypoxia frequently result in impaired mitochondrial biogenesis, primarily attributed to the transcriptional suppression of the key coactivator, peroxisome proliferator‐activated receptor‐gamma coactivator 1‐alpha (PGC‐1α). Mechanistic studies by Scharping et al. revealed that PGC‐1α deficiency serves as a central driver of metabolic insufficiency and exhaustion in TILs [41]. Building upon this mechanism, Lontos et al. recently developed engineered chimeric antigen receptor (CAR) T cells overexpressing PGC‐1α. This modification not only restored mitochondrial mass and morphology but also significantly enhanced the spare respiratory capacity (SRC) of T cells, enabling them to flexibly utilize FAO for survival in glucose‐restricted environments [207]. Such metabolic reprogramming strategies significantly augment the persistent anti‐tumor efficacy of CAR‐T cells within the harsh TME.

### 8.3 PD‐1 and Drp1

The inhibitory effect of PD‐1 on T cells is not confined to proximal TCR signaling but also profoundly influences mitochondrial morphodynamics. Research by Simula et al. revealed that PD‐1 signaling suppresses the ERK1/2 and mTOR pathways, thereby blocking the activating phosphorylation of the mitochondrial fission protein Drp1 at the Thr616 residue. This leads to aberrant mitochondrial fission, manifesting as excessively elongated, fused mitochondrial structures accompanied by compromised cristae integrity [51]. Subsequent investigations by Ogando et al. further confirmed that blockade of the PD‐1/PD‐L1 axis can rapidly restore Drp1 activity and the equilibrium of mitochondrial dynamics, which is critical for the elimination of damaged mitochondria and for facilitating the high‐efficiency migration of Teffs within the tumor stroma [208].

### 8.4 CTLA‐4

Distinct from PD‐1, which primarily modulates mitochondrial function, CTLA‐4 is regarded as the quintessential glycolytic checkpoint of T cells. Pivotal research by Frauwirth et al. elucidated that CTLA‐4, through competitive binding to B7 molecules, inhibits the CD28‐PI3K‐Akt signaling axis, thereby directly downregulating the expression of glucose transporter 1 (Glut1) and the activity of key glycolytic enzymes [209]. Subsequent investigations by Patsoukis et al. further delineated the divergent metabolic impacts of these checkpoints: while CTLA‐4 predominantly suppresses glycolysis while preserving a degree of OXPHOS capacity, PD‐1 alters metabolic substrate preference by promoting fatty acid *β*‐oxidation [17].Consequently, the essence of CTLA‐4 blockade therapy lies in alleviating the metabolic constraints on the T‐cell Warburg effect, thereby satisfying the heightened anabolic requirements essential for the rapid proliferative phase of anti‐tumor immunity.

### 8.5 Atg5

Autophagy plays a dual role in Teffs, but the prevailing consensus highlights its critical importance for maintaining cellular fitness. Utilizing an Atg5 conditional knockout model, DeVorkin et al. demonstrated that autophagy is a prerequisite for the survival of CD8^+^ Teffs within the TME. The loss of Atg5 leads to the accumulation of dysfunctional mitochondria and ROS, triggering metabolic collapse and apoptosis, thereby compromising anti‐tumor immunity [210]. Furthermore, Puleston et al. indicated that autophagic flux is indispensable for the formation and long‐term persistence of memory T cells (Tmem) [211]. Consequently, strategies targeting Teffs should focus on the pharmacological activation of autophagy to enhance organellar quality control.

### 8.6 Atg7 and Vps34

In contrast to its protective role in Teffs, autophagy serves a distinct function in maintaining the lineage stability and suppressive capacity of Tregs. Wei et al. demonstrated that Tregs are exquisitely sensitive to the deficiency of autophagy‐related genes; the genetic ablation of Atg7 or Vps34 precipitates severe metabolic derangement and apoptosis in Tregs within the TME, thereby leading to a catastrophic loss of their immunosuppressive function [212]. Further research by Willinger et al. confirmed that pharmacological blockade of the autophagic pathway can selectively attenuate Treg‐mediated suppression while simultaneously maintaining or even augmenting the anti‐tumor efficacy of Teffs [213]. These findings suggest that the targeted inhibition of autophagy to compromise Treg fitness represents a promising therapeutic avenue for remodeling the tumor immune microenvironment (TIME) and overcoming immune evasion.

### 8.7 Flcn

Nutrient sensing on the lysosomal surface serves not only as a metabolic regulator but also as a pivotal checkpoint for T‐cell differentiation. Recent investigations by Raynor et al. established a central negative regulatory role for folliculin (Flcn) in this process. Flcn typically functions as a GTPase‐activating protein (GAP) for RagC, facilitating mTORC1 activation and the cytoplasmic sequestration of TFEB. Research demonstrated that while Flcn deficiency suppresses potent mTORC1‐driven glycolytic signaling, it relieves the inhibition on TFEB, enabling its nuclear translocation and the initiation of an adaptive program characterized by enhanced lysosomal biogenesis and lipid metabolism [214]. This distinct metabolic remodeling selectively favors the survival and differentiation of TRM, endowing them with enhanced long‐term persistence and anti‐infective protective capacity within tissues such as the intestine. These findings highlight Flcn as a novel target for enhancing mucosal immunological memory through metabolic intervention.

### 8.8 IRE1α

Under persistent antigenic stimulation, T cells encounter a substantial protein synthesis burden, leading to the chronic activation of the UPR. Song et al. discovered that the ER stress sensor IRE1α is overactivated in TILs, where its RNase domain degrades an array of mRNAs critical for cell survival via RIDD. Utilizing small‐molecule kinase inhibitors to allosterically inhibit IRE1α oligomerization can specifically block deleterious RIDD activity while preserving the splicing of spliced XBP1s, which is indispensable for mitochondrial function and the expression of effector molecules [98]. This strategy precisely redirects the ER stress response from a pro‐apoptotic toward a pro‐survival trajectory.

### 8.9 BiP, PDI, and Cholesterol

The protein folding capacity of the ER serves as a rate‐limiting factor for the secretion of T‐cell effector molecules. Ma et al. demonstrated that elevated cholesterol levels within the TME augment the rigidity of the T‐cell ER membrane, thereby interfering with the chaperone functions of BiP and protein disulfide isomerase (PDI); this precipitates the misfolding of immune checkpoint molecules and cytokines, which ultimately exacerbates proteotoxic stress and terminal dysfunction [106]. Conversely, Yang et al. proposed that the inhibition of ACAT1, a cholesterol esterification enzyme, increases the levels of free cholesterol within the plasma membrane. This modification not only facilitates TCR nanoclustering and enhances signal transduction but also promotes ER proliferation and functional capacity through feedback mechanisms, significantly bolstering the anti‐tumor activity of CD8^+^ T cells [108].

### 8.10 c‐Jun

Transcription factor imbalance is a critical underlying factor in T‐cell exhaustion. Pivotal research by Lynn et al. revealed that c‐Jun functional deficiency in Terminal Tex leads to insufficient formation of AP‐1 complexes, causing NFAT to primarily bind DNA in an unpaired form, thereby initiating the transcription of exhaustion‐related genes such as TOX and PD‐1. Overexpressing c‐Jun through genetic engineering can restore functional c‐Jun/c‐Fos dimer formation and competitively relocate NFAT to immune activation‐related gene loci [215]. This transcriptional remodeling endows CAR‐T cells with significant anti‐exhaustion characteristics, enabling them to maintain chromatin accessibility and effector functions under long‐term antigen exposure.

### 8.11 ACAT1

Cholesterol is not only a structural component of the cell membrane but also a key regulatory molecule for T‐cell signal transduction. ACAT1 is the critical rate‐limiting enzyme that catalyzes the esterification and storage of free cholesterol. Pioneering research by Yang et al. revealed that ACAT1 serves as a metabolic checkpoint for CD8^+^ T cells; inhibiting ACAT1 activity through small‐molecule inhibitors or genetic knockout leads to the intracellular accumulation of free cholesterol and significantly elevates plasma membrane cholesterol content. This alteration in membrane lipid composition facilitates TCR nanoclustering at the IS, thereby lowering the antigen recognition threshold and enhancing downstream signal transduction efficiency. Furthermore, the elevation of plasma membrane cholesterol synergistically promotes T‐cell proliferation as well as the synthesis and release of cytotoxic granules. Notably, although excessive cholesterol within the TME may induce ER stress, the ACAT1 inhibition strategy effectively improves the effector function of CD8^+^ T cells by specifically enhancing plasma membrane signaling. Research has confirmed that this intervention significantly bolsters the capacity of CD8^+^ T cells to control the growth of cutaneous melanoma and produces a notable synergistic anti‐tumor effect when combined with anti‐PD‐1 therapy [108].

### 8.12 Helios

Helios is a member of the Ikaros transcription factor family, playing a pivotal role in maintaining T‐cell anergy and Treg stability. Research by Kim et al. demonstrated that Helios leads to epigenetic silencing of the Il2 locus by recruiting HDACs to its promoter region. In exhausted T cells or unstable Tregs, targeted degradation or knockout of Helios can rapidly rescind this inhibition, restoring histone acetylation levels and interleukin‐2 (IL‐2) transcription [216]. Currently, the targeted degradation of Ikaros family proteins using thalidomide‐like drugs or proteolysis‐targeting chimera (PROTAC) technology has emerged as a preclinical research direction for reversing T‐cell transcriptional repression.

### 8.13 Lamin A/C

In high‐stiffness solid tumors such as PDAC, T cells encounter the risk of nuclear mechanical damage during interstitial migration. Nader et al. discovered that the physical compression of the nucleus can lead to nuclear envelope rupture; this not only induces DNA damage but also permits the entry of the nuclease TREX1 into the nucleus to degrade genomic DNA, thereby triggering genomic instability and cell death [217]. However, as noted in Section 5.1.1, statically maintaining or augmenting Lamin A/C expression imposes a structural constraint, wherein the loss of nuclear deformability irreversibly impedes T‐cell interstitial migration. Therefore, optimizing T‐cell mechanical resilience depends on dynamic, context‐specific mechanical regulation rather than stable overexpression. Emerging bioengineering strategies may utilize mechanosensitive promoters (e.g., Piezo1‐driven circuits) to transiently upregulate Lamin A/C solely during peak nuclear compression within dense matrices, followed by rapid downregulation upon entering looser microenvironments. This dynamic calibration uncouples nuclear protection from migratory arrest, enabling T cells to safely navigate the physical barriers of solid tumors (Table 1).

**TABLE 1:** Summary of Therapeutic Targets and Mechanisms for Reversing T Cell Exhaustion Based on Subcellular Intervention.

Targeted Organelle	Therapeutic Target	Regulatory Strategy & Mechanism	Metabolic & Subcellular Effect	Effector Cell Type	Functional Outcome
Mitochondria	MCJ	Gene silencing / Deficiency	Enhanced mitochondrial respiration & supercomplex assembly	CD8^+^ T Cell	Relieves electron transport chain constraints, significantly enhancing antiviral/anti‐tumor immunity
	PD‐1	Antibody blockade	Regulates Drp1‐dependent fission	Intratumoral T Cell	Reverses abnormal mitochondrial morphology, restoring T‐cell migration and proliferation capacity
	CTLA‐4	Antibody blockade	Restores glucose metabolism blocked by CD28 inhibition	CD4^+^ T Cell	Enhances T‐cell function by restoring the metabolic activity of the CD28 signaling pathway
	PGC‐1α	Gene overexpression	Increased mitochondrial biogenesis & SRC	CAR‐T Cell	Maintains metabolic fitness in the solid TME, enhancing anti‐tumor persistence
Lysosome / Autophagy	Atg5	Autophagy regulation	Maintenance of mitochondrial quality via autophagy	CD8^+^ Effector T	Autophagy is essential for effector T‐cell survival; autophagic integrity supports anti‐tumor immunity
	Atg7 / Vps34	Gene deletion	Coupling environmental cues to metabolism	Tregs	Leads to the loss of functional integrity of intratumoral Tregs, relieving immunosuppression
	Flcn	Phosphorylation regulation	Insulin‐mediated mTORC1 activation	T Cell / General	Regulates the mTORC1 signaling pathway, affecting cell growth and metabolic processes
ER	IRE1α	Kinase inhibitor (e.g., KIRA6)	Inhibits oligomerization, spares XBP1s	T Cell	Blocks ER stress‐induced exhaustion, maintaining effector functions
	BiP / PDI	Chemical chaperone (TUDCA)	Relieves UPR, reduces cholesterol burden	TILs	Restores correct folding and secretion of IL‐2/IFN‐γ, alleviating proteotoxic dysfunction
	ACAT1	Enzyme inhibition	Increases plasma membrane cholesterol	CD8^+^ T Cell	Enhances TCR signaling and IS formation, boosting cytolytic activity
Nucleus	c‐Jun	Gene overexpression	Restores functional AP‐1 heterodimers	CAR‐T Cell	Blocks the exhaustion gene program, endowing cells with exhaustion‐resistant traits
	Helios	Degradation / Knockout	Relieves suppression of Il2 promoter	Treg / Exhausted T	Restores IL‐2 production, promoting T‐cell conversion to an effector phenotype
	Lamin A/C	Structural maintenance	Balances nuclear deformability and integrity; prevents rupture without arresting migration	Infiltrating T	Prevents DNA damage and genomic instability, promoting tumor infiltration

## 9 Subcellular‐Targeted Therapeutic Intervention Strategies for Cancer

Addressing the multi‐tiered subcellular dysfunctions involved in T‐cell exhaustion, traditional pharmacological approaches often struggle to effectively traverse cellular and organellar membrane barriers due to insufficient targeting and limited bioavailability. Emerging immuno‐engineering strategies based on functional nanomaterials provide novel possibilities for the precise repair of organelles and the remodeling of T‐cell functions at the subcellular scale. This section explores how technologies such as nanozymes, smart delivery vehicles, and organelle transplantation can be utilized to restore the metabolic adaptability and anti‐tumor efficacy of T cells from a subcellular perspective.

### 9.1 Regulation of Mitochondrial Function

Mitochondria serve as a central nexus in T cell exhaustion, and their functional restoration primarily depends on scavenging excessive ROS to preserve organelle integrity and reprogramming metabolic flux to reinstate efficient OXPHOS. Translating these mechanistic insights into clinically viable therapies, however, confronts substantial bioengineering hurdles. Although modulation of mitochondrial dynamics has demonstrated preclinical rationale, the principal challenge resides in the precision of systemic delivery—specifically, ensuring that interventions effectively reach tumor‐infiltrating lymphocytes embedded within the dense stromal architecture of the TME without premature sequestration by physiological barriers.

#### 9.1.1 Nanozymes for ROS Scavenging

To counter the persistent ROS stress within the solid tumor microenvironment, nanozymes with high stability and multienzymatic catalytic activities have emerged as a preclinical alternative to natural antioxidant enzymes [218]. Cerium oxide nanoparticles, through their mixed Ce3+/Ce4+ valence states, mimic superoxide dismutase and catalase activities, effectively preserving mtDNA and mitochondrial membrane potential [219]. Similarly, manganese dioxide nanomaterials alleviate oxidative stress in T cells by generating oxygen and downregulating HIF‐1α, thereby relieving hypoxia‐mediated suppression of mitochondrial biogenesis [220, 221, 222]. Prussian blue nanoparticles also exhibit broad‐spectrum ROS scavenging capacity and may delay terminal exhaustion through modulation of mitochondrial signaling pathways such as p38 MAPK [223, 224].

The in vivo feasibility of these nanoscavengers, however, is constrained by translational hurdles. A major impediment to systemically administered nanozymes is sequestration by the reticuloendothelial system, which reduces the effective concentration reaching the intratumoral TIL pool. Moreover, introduction of inorganic nanoparticles may provoke systemic innate immune responses or acute inflammation. Future efforts must concentrate on sophisticated surface modifications, such as biomimetic coating or functionalization with T cell‐specific ligands, to facilitate RES evasion and ensure targeted delivery to TILs while maintaining a robust safety profile.

#### 9.1.2 Targeted Delivery of Metabolic Modulators

Although metabolic modulators such as NAD^+^ precursors or metformin have demonstrated considerable potential in reversing exhaustion hallmarks in vitro, their in vivo efficacy is frequently constrained by unfavorable pharmacokinetic profiles and nonspecific distribution. To circumvent these obstacles, functionalized nanocarriers have been extensively deployed for preclinical proof‐of‐concept. For instance, encapsulation of NAD^+^ precursors within liposomes surface‐modified with anti‐CD3 or anti‐CD8 antibodies is designed to actively target T cells, promoting lysosomal escape to activate the Sirt1/PGC‐1α mitochondrial biogenesis axis [225, 226]. Concurrently, pH‐responsive MOFs [227] and albumin nanoparticles delivering A2A receptor antagonists [228] are engineered to achieve spatiotemporally controlled release within the acidic and adenosine‐rich TME. Furthermore, the research community has explored biomimetic cell membrane‐camouflaged carriers for the co‐delivery of the mitochondrial fission inhibitor Mdivi‐1 and anti‐PD‐1 antibodies, seeking synergistic restoration of structural and functional integrity.

These active targeting systems, however, differ fundamentally from passive nanocarriers. Antibody‐functionalized constructs are susceptible to the peripheral sink effect, undergoing premature consumption by circulating or lymphatic naïve T cells prior to reaching the tumor bed. Even upon entry into the TME, the binding site barrier restricts high‐affinity carriers to the tumor periphery, impeding penetration through the dense stroma to reach deeply infiltrated terminally exhausted T cells. Moreover, delivery of substrates such as NAD^+^ carries inherent risks of metabolic nonspecificity, as premature leakage within the TME may inadvertently provide energetic support to malignant cells or immunosuppressive stroma. Additionally, the multicomponent nature of biomimetic camouflaged architectures introduces complex chemistry, manufacturing, and control challenges, rendering clinical scale‐up profoundly difficult.

#### 9.1.3 Mitochondrial Transplantation and Artificial Mitochondrial Technology

When endogenous mitochondria in Terminal Tex cells sustain irreversible structural damage, mitochondrial replacement therapy emerges as a conceptual salvage strategy. In vitro studies demonstrate that functional mitochondria can be transferred into exhausted T cells via MSC‐derived tunneling nanotubes [229, 230] or delivered directly as isolated autologous or allogeneic mitochondria using cell‐penetrating peptides [231]. Furthermore, investigators have envisioned synthetic mitochondria, biomimetic liposomal microreactors loaded with ATP synthase, to provide stable energetic support independent of biological donors [232].

However, specifically targeting these structures to TILs in vivo confronts formidable physiological and immunological barriers. Mitochondria or their synthetic surrogates, given their substantial dimensions (0.5 to 1.0 µm), are almost entirely sequestered by the RES in the liver and spleen following systemic administration, rendering penetration of the dense tumor stroma exceedingly difficult. More critically, exogenous mitochondria constitute a source of DAMPs, capable of releasing unmethylated CpG mtDNA and formylated peptides. Upon intravenous injection, this readily triggers systemic innate immune cascades via TLR9 or cGAS‐STING pathways, potentially precipitating inflammatory storms. Consequently, pending resolution of these pharmacokinetic and immunological paradoxes, organelle transplantation remains a feasible strategy primarily as an ex vivo preconditioning maneuver for adoptive cell therapy rather than a direct in vivo intervention.

### 9.2 Restoration of ER Homeostasis and Protein Folding

The ER of Terminal Tex cells frequently exhibits structural dilation and compromised folding capacity, rendering endogenous UPR insufficient to maintain proteostasis. To counteract this, targeted engineering strategies aim to deliver exogenous chaperones or modulate UPR signaling nodes, although translating such interventions in vivo confronts unique pharmacological obstacles.

#### 9.2.1 Targeted Delivery of ER Stress Alleviators

Small‐molecule chemical chaperones, such as 4‐phenylbutyrate (4‐PBA) and tauroursodeoxycholic acid (TUDCA), can alleviate ER stress by stabilizing unfolded proteins. Given that these compounds require millimolar concentrations and exhibit short plasma half‐lives, functionalized nanocarriers have been deployed as preclinical proof‐of‐concept delivery vehicles [233]. For instance, liposome‐encapsulated TUDCA has been shown to downregulate ER stress markers GRP78 and CHOP in murine melanoma models [106]. Similarly, conjugating 4‐PBA to a hyaluronic acid backbone yields self‐assembling nanogels designed to target CD44 receptors, enabling chaperone enrichment proximal to the ER to facilitate MHC‐I assembly and antigenic peptide loading [234].

However, advancing these nanoscale interventions toward clinical application encounters translational challenges. On one hand, passive accumulation reliant on the enhanced permeability and retention effect is constrained in human solid tumors, as elevated interstitial fluid pressure and dense stroma severely impede nanoparticle extravasation. On the other hand, active targeting utilizing receptors such as CD44 suffers from receptor nonspecificity. CD44 is ubiquitously overexpressed on tumor cells, CAFs, and TAMs, leading to substantial sequestration of hyaluronic acid nanogels by the tumor parenchyma and immunosuppressive stroma. This creates an intratumoral biological sink that prevents effective doses from reaching Terminal Tex cells. Consequently, achieving T cell‐specific restoration of ER proteostasis necessitates circumventing these off‐target cellular sinks.

#### 9.2.2 Unfolded Protein Response Modulators

To counter the hyperactivated IRE1α‐XBP1 axis in Terminal Tex cells, the small‐molecule inhibitor KIRA6 has demonstrated considerable mechanistic potential. It can allosterically inhibit IRE1α oligomerization, aiming to selectively block the pro‐apoptotic RIDD activity while preserving the adaptive splicing function of XBP1s [235, 236]. Given the pronounced hydrophobicity and potential systemic toxicity of KIRA6, functionalized nanocarriers have been extensively utilized as preclinical delivery tools [237]. For instance, pH‐responsive polymeric micelles such as PDPA or polyhistidine are engineered to undergo structural dissociation and rapid drug release within the acidic TME [238]. Furthermore, acid‐sensitive albumin nanoparticles have been developed to deliver KIRA6 to tumor‐draining lymph nodes, with the goal of intercepting ER stress signaling during the early stages of T cell priming [239].

However, the clinical application of these UPR‐modulating platforms confronts multiple equipoise challenges. Acid‐triggered release lacks cellular selectivity, readily exposing adjacent dendritic cells and tumor cells to KIRA6, thereby potentially perturbing antigen presentation or inadvertently alleviating ER stress in malignant cells. Simultaneously, the UPR exhibits bidirectional functionality: transient IRE1α inhibition may confer protection to T cells, whereas sustained suppression impairs ER expansion capacity and compromises effector protein secretion. Consequently, systemic UPR modulation remains a high‐risk endeavor pending the attainment of T cell‐specific targeting and precise spatiotemporal control.

#### 9.2.3 mRNA‐Based Delivery of Folding Chaperones

Beyond small‐molecule modulation, direct supplementation of ER‐resident chaperones represents a genetic strategy to augment the protein folding capacity of T cells. By optimizing the pKa of ionizable cationic lipids within LNPs to enhance endosomal escape, delivery of mRNA encoding BiP or PDI into the T cell cytosol has become feasible [240, 241]. Overexpressed BiP prevents aberrant polypeptide aggregation, while PDI facilitates correct disulfide bond formation, a process critical for the folding of disulfide‐rich effector molecules such as IL‐2, IFN‐γ, and perforin [106, 242]. Preclinical investigations indicate that such mRNA‐mediated reinforcement can alleviate the protein secretory burden of T cells during rapid proliferative expansion [243].

However, this strategy encounters kinetic mismatch. T cell exhaustion is a chronic process, whereas mRNA expression is transient, and brief elevation of chaperone levels is unlikely to confer durable protection. Moreover, repeated LNP administration carries inherent risks of lipid toxicity and immunogenicity. Transient expression is therefore better suited as an ex vivo preconditioning maneuver for CAR‐T cell manufacturing, endowing T cells with a temporary ER stress buffer during preparation to enhance survival and effector output during the early phase of tumor engraftment. To achieve sustained in vivo efficacy, future approaches will necessitate stable genomic integration of ER regulatory circuits via lentiviral vectors or CRISPR, thereby aligning therapeutic intervention with the chronic temporal scale of T cell stress.

### 9.3 Nuclear and Epigenetic Interventions

A central mechanism underlying terminal exhaustion resides in the irreversible remodeling of the epigenetic landscape driven by transcription factors such as TOX and the NR4A family. To circumvent the genomic damage risks associated with conventional double‐strand break gene editing, emerging strategies focus on sequence‐agnostic approaches, including precise epigenetic editing and targeted protein degradation. These subcellular nuclear interventions aim to reprogram transcriptional states and restore functional plasticity in antitumor T cells.

#### 9.3.1 Epigenetic Editing

To specifically silence exhaustion‐driving transcription factors without altering DNA sequence, CRISPR‐dCas9‐based epigenetic editors have emerged as high‐specificity proof‐of‐concept tools [244]. By fusing catalytically inactive dCas9 with transcriptional repression domains, these systems can deposit repressive histone modifications at promoter regions of TOX or NR4A [245, 246]. To deliver these macromolecular complexes, investigators commonly employ engineered lipid nanoparticles to transport mRNA encoding dCas9 and single guide RNA in preclinical models [240, 247].

However, durable single‐dose silencing in vivo confronts multiple practical obstacles. Resting and exhausted T cells are highly refractory to LNP transfection, and even upon successful endosomal escape, the bulky dCas9 complex faces considerable difficulty traversing the stringent nuclear pore complex. Artificially deposited histone modifications are susceptible to dilution during rapid T cell proliferation, leading to epigenetic erosion. Moreover, indiscriminate silencing of TOX, while capable of suppressing terminal exhaustion, may also ablate critical safeguards that prevent AICD and preserve the progenitor pool, thereby carrying the risk of therapeutic overshoot.

#### 9.3.2 Targeted Degradation of Transcription Factors

Beyond transcriptional repression, direct degradation of intracellularly accumulated exhaustion‐associated transcription factors utilizing PROTACs or molecular glues constitutes an alternative interventional avenue. These bifunctional molecules simultaneously engage the target protein and an E3 ubiquitin ligase, thereby inducing target ubiquitination and subsequent proteasomal degradation. Given their substantial molecular weight and poor membrane permeability, nanocarrier‐mediated delivery is critical for enhancing the bioavailability of PROTACs. A more advanced design involves the construction of TME‐activated prodrug nanoparticles, wherein elevated glutathione or ROS levels trigger cleavage of labile linkers to locally release the active PROTAC molecule, thereby improving cellular entry efficiency and tumor selectivity [248]. Investigations have demonstrated that nanocarrier‐mediated delivery of a Helios‐targeting PROTAC can rapidly diminish nuclear Helios levels, relieve its transcriptional repression of promoters such as IL‐2, and transiently restore T cell proliferative and secretory capacity, achieving rapid functional reinvigoration [216].

However, the in vivo translation of these TME‐responsive PROTAC nanosystems confronts a dual paradox of pharmacokinetics and cellular uptake. On one hand, exhausted T cells exhibit endocytic resistance, and systemically administered nanoparticles are predominantly sequestered by phagocytic stroma, rendering efficient transfection of intratumoral T cells exceedingly difficult. On the other hand, TME‐triggered nanocarrier dissociation releases PROTAC molecules prematurely into the extracellular space, and given their extremely poor membrane permeability, these agents cannot traverse the plasma membrane to access the cytosol and nucleus for functional engagement. Consequently, pending the achievement of T cell‐autonomous cytosolic delivery, PROTAC technology should be primarily confined to ex vivo engineering of adoptive cell therapies, thereby circumventing these in vivo trafficking impediments.

#### 9.3.3 Extracellular Matrix Regulation to Improve Nuclear Function

In highly desmoplastic solid tumors, mechanical compression imposed by the dense ECM constitutes a formidable physical barrier that can precipitate nuclear envelope rupture and genomic instability in T cells [217]. To alleviate mechanical stress, investigators have developed matrix‐targeted nanocarriers loaded with matrix‐degrading enzymes as stimuli‐responsive delivery systems [249]. Theoretically, localized stromal relaxation could preserve nuclear integrity by mitigating extreme deformation during T cell migration, and could indirectly safeguard DNA repair mechanisms through improved microvascular perfusion and relief of hypoxia.

**FIGURE 8:**
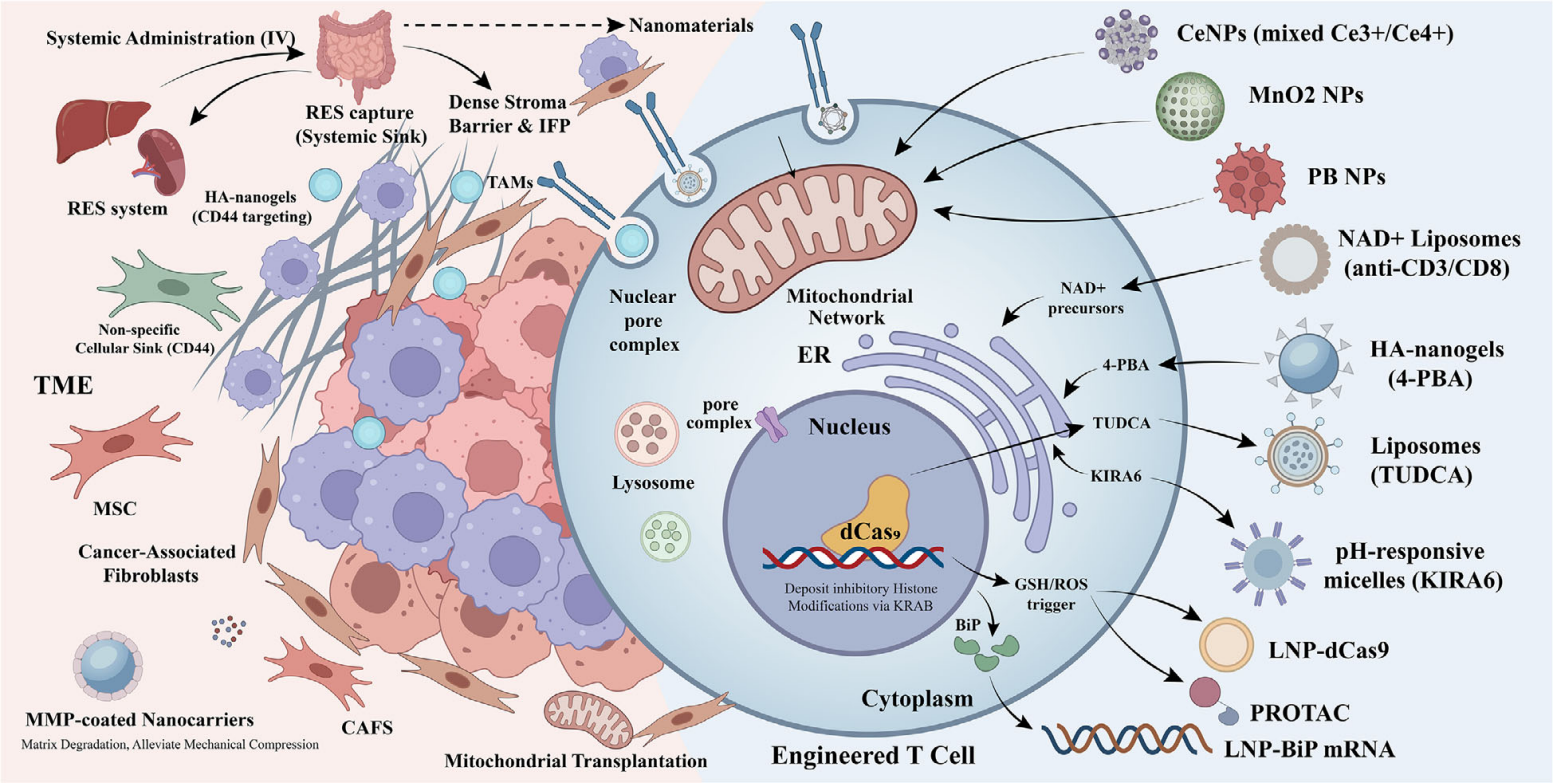
Exogenous pharmacological and nanomedicine intervention strategies targeting subcellular homeostasis in T cells.

However, matrix‐degrading interventions confront numerous clinical dilemmas. Although the dense stroma impedes T cell infiltration, it also serves as a physical barrier constraining tumor dissemination. Uncalibrated enzymatic degradation may dismantle this barrier, thereby inadvertently opening conduits for metastatic escape. Moreover, broad stromal depletion in tumors such as PDAC has been shown to accelerate disease progression and enrich immunosuppressive cell populations. Consequently, unless stromal remodeling can be strictly confined to the pericellular space surrounding migrating T cells, such interventions remain a high‐risk clinical proposition (Figure 8).

### 9.4 Next‐Generation Cell Therapies

Conventional ACT has primarily focused on optimizing antigen recognition receptors, yet the current research frontier is shifting toward systematic engineering of intrinsic subcellular functions within T cells. By rewiring their transcriptional networks, metabolic pathways, and stress response systems through synthetic biology approaches, the goal is to construct functionally enhanced T cells that are intrinsically resistant to exhaustion. However, integrating these multifaceted cellular circuits confronts challenges pertaining to genetic payload capacity and manufacturing compliance.

#### 9.4.1 Transcriptional and Organellar Reinforcement

To address the critical functional loss of AP‐1 complexes in exhausted T cells, studies have demonstrated that genetic engineering of T cells to stably express the transcription factor c‐Jun can effectively restore the formation of functional AP‐1 complexes. The reconstituted c‐Jun/Fos dimers competitively displace inhibitory factors bound to NFAT, thereby blocking the transcriptional initiation of exhaustion‐driving genes including TOX and PDCD1 [215, 250]. Concurrently, to counteract the energetic deprivation within the TME, introduction of the master regulator of mitochondrial biogenesis, PGC‐1α, has been shown to significantly enhance the spare respiratory capacity of CAR‐T cells, enabling efficient utilization of alternative fuels such as fatty acids [207]. Furthermore, to alleviate the protein folding burden associated with rapid proliferation, co‐expression of ER‐resident chaperones such as BiP or PDI represents a rational strategy to prevent lethal proteotoxicity [251].

However, these subcellular strategies confront numerous clinical bottlenecks. Existing clinical‐grade lentiviral or retroviral vectors are limited in their capacity to safely and efficiently accommodate the CAR construct alongside multiple large genetic cassettes such as c‐Jun, PGC‐1α, and ER chaperones simultaneously. From a safety perspective, sustained forced mitochondrial biogenesis and the overriding of fundamental transcriptional checkpoints such as NFAT/TOX may precipitate replicative senescence in T cells or, through excessive activation, lead to malignant transformation. Consequently, the transition from single‐gene enhancement to multiplexed subcellular engineering will depend on breakthroughs in nonviral site‐specific integration technologies and must be accompanied by rigorous long‐term in vivo safety assessments to prevent the therapeutic T cells themselves from acquiring tumorigenic potential.

#### 9.4.2 Environment Sensing and Signal Conversion

Beyond enhancing intrinsic cellular resilience, endowing T cells with synthetic environmental sensing circuits aims to actively convert suppressive TME signals into activation cues. For instance, fusing the extracellular domain of PD‐1 with the intracellular signaling domain of CD28 yields a chimeric switch receptor. Upon encountering PD‐L1 on the tumor surface, these engineered CAR‐T cells can theoretically transduce an inhibitory checkpoint signal into a CD28 costimulatory signal [252, 253]. Similarly, coupling pH‐ or lactate‐sensing domains with downstream regulatory elements can conditionally restrict potent CAR‐T activation to acidic tumor regions [254].

However, the clinical application of these synthetic sensing circuits confronts the dual challenges of sustained signal transduction and spatial heterogeneity. On one hand, persistent PD‐L1‐driven CD28 signaling within the TME can precipitate metabolic overexertion of T cells, accelerating their progression toward activation‐induced cell death or terminal exhaustion. On the other hand, circuits designed around acidity or lactate are constrained by the markedly uneven intratumoral pH gradient: excessively stringent thresholds render them ineffective at the tumor periphery, whereas overly permissive thresholds incur off‐target toxicity. Consequently, achieving dynamic and safe circuit calibration remains a preclinical engineering challenge that demands urgent resolution.

## 10 Anti‐Tumor Therapeutic Strategies Based on Organelle Interactions

While single‐organelle targeting strategies have demonstrated promise in preclinical models, they frequently encounter resistance challenges within the intricate TME. Organelles serve not only as the effectors of T‐cell metabolic adaptation but also as central hubs for cancer cells to navigate therapeutic pressure. Therefore, the integrated use of organelle modulators with existing anti‐angiogenic therapies, radiotherapy, chemotherapy, and novel ICIs aims to achieve synergistic anti‐tumor effects through organelle crosstalk and interaction.

### 10.1 Combination With Anti‐Angiogenic Drugs

Structural abnormalities in tumor vasculature lead to severe localized hypoxia and acidosis within the TME; hypoxia directly inhibits the expression of PGC‐1*a* in T cells, resulting in impaired mitochondrial biogenesis and forcing T cells into a state of metabolic insufficiency. Utilizing anti‐angiogenic drugs such as bevacizumab or small‐molecule tyrosine kinase inhibitors (TKIs) can achieve vascular normalization rather than simple vascular destruction. Research by Huang et al. confirmed that low‐dose anti‐angiogenic therapy can prune immature vessels, improving tumor perfusion and oxygen supply. Reoxygenation of the TME directly relieves the inhibition on T‐cell mitochondria, thereby restoring their OXPHOS capacity and IFN‐γ production. When used in combination with PGC‐1*a* agonists or mitochondrial enhancers such as bezafibrate, these metabolic reprogramming effects can be further consolidated, establishing a positive feedback loop that promotes anti‐tumor function [255].

### 10.2 Combination With Chemotherapy

Traditional chemotherapeutic agents not only directly eliminate tumor cells by inducing DNA damage but also initiate anti‐tumor immune responses by triggering ER stress. For instance, doxorubicin or oxaliplatin can induce immunogenic cell death (ICD) in tumor cells, a process dependent on the *PERK‐eIF2α*‐mediated ER stress pathway. Upon activation, this pathway promotes the translocation of calreticulin (CRT) from the ER lumen to the cell surface. Surface‐exposed CRT is recognized by the CD91 receptor on dendritic cells (DCs), thereby enhancing antigen presentation and facilitating the recruitment of CTLs. As emphasized by Galluzzi et al., combining these ER stress‐inducing chemotherapeutics with ICB can transform previously non‐responsive T cells within the TME into cells with robust anti‐tumor activity [256].

### 10.3 Combination With Radiotherapy

Radiotherapy‐induced DNA DSBs lead to the formation of micronuclei in tumor cells; due to the extreme fragility of the nuclear envelope, these micronuclei are prone to rupture, resulting in the leakage of double‐stranded DNA (*dsDNA*) into the cytoplasm. Cytoplasmic *dsDNA* is recognized by the DNA sensor *cGAS*, which activates the *STING‐IRF3* signaling pathway and induces the explosive secretion of Type I interferons (*IFN‐I*). This cytokine milieu can effectively reverse the phenotype of exhausted T cells. However, research published by Vanpouille‐Box et al. revealed that the selection of radiotherapy dosage is paramount, as excessively high doses induce the expression of the exonuclease TREX1, which degrades cytoplasmic DNA and subsequently silences the *cGAS* pathway. Therefore, adopting hypofractionated radiotherapy regimens in combination with TREX1 inhibitors or nuclear envelope stabilizers represents a frontier strategy for maximizing T‐cell recruitment and activation [257].

### 10.4 Combination With TGF‐*β*

TGF‐*β* serves as a critical barrier driving immunotherapy resistance within the TME; TGF‐*β* signaling potently blocks the release of Type I interferons (*IFN‐I*) within the tumor milieu. *IFN‐I* constitutes the essential “third signal” required for the priming, survival, and execution of cytotoxic functions in *CD8^+^* T cells. The absence of this critical signal results in the inability of T cells to effectively infiltrate or maintain their cytolytic activity within the tumor, ultimately contributing to the failure of spontaneous tumor rejection. This microenvironmental signaling inhibition is closely associated with the failure of monotherapeutic ICB. The combined use of TGF‐*β* neutralizing antibodies or inhibitors can rescind the suppression of *IFN‐α/β* secretion, thereby remodeling a cytokine microenvironment conducive to T‐cell function. The restored *IFN‐I* signaling not only promotes tumor antigen presentation by DCs but also directly augments the effector programs of *CD8^+^* T cells, enabling them to re‐recognize and eliminate tumor cells. This strategy significantly enhances the anti‐tumor efficacy of immunotherapy by rebuilding a positive feedback loop between the microenvironment and T cells [258].

### 10.5 Targeting MDSCs

MDSCs are pivotal regulatory cells that drive T‐cell mitochondrial dysfunction within the TME. MDSCs utilize high expression of fatty acid transport protein 2 (FATP2) for the massive uptake of lipids, which are not only utilized for their own oxidative metabolism but also directly suppress T‐cell function through the release of mediators such as prostaglandin E2 (*PGE_2_*). Research by Veglia et al. has demonstrated that targeted inhibition of FATP2 can block the metabolic reprogramming of MDSCs, thereby reversing their immunosuppressive activity. Further investigation revealed that inhibiting the metabolic uptake function of MDSCs restores arginine and glucose levels within the TME, alleviating the nutrient‐deprived state of T cells and subsequently normalizing their mTORC1 signaling and mitochondrial function. The combination of FATP2 inhibitors with ICB yields significant synergistic anti‐tumor efficacy based on this mechanism [259].

### 10.6 Combination With Oncolytic Viruses

Oncolytic viruses (OVs) not only directly lyse tumor cells but also serve as potent organelle stress inducers. Viral infection rapidly triggers the massive generation of mitochondrial *ROS* and simultaneously induces the *ER* stress response. When utilized as in situ vaccines, the organelle damage caused by OVs releases a plethora of DAMPs—such as *ATP* and high mobility group box 1 (*HMGB1)*—alongside viral antigens. Research by Bommareddy et al. has demonstrated that the microenvironment shaped by such organelle stress exerts a natural immune adjuvant effect, significantly enhancing the activation of antigen‐presenting cells (APCs). Furthermore, by engineering OVs to express metabolic modulators such as *IL‐15/IL‐21* super‐agonists or leptin, it is possible to provide mitochondrial functional support signals for infiltrating T cells while simultaneously lysing the tumor, thereby preventing functional exhaustion during the anti‐tumor immune response [260].

### 10.7 Combination With Next‐Generation Checkpoint Inhibitors

**FIGURE 9:**
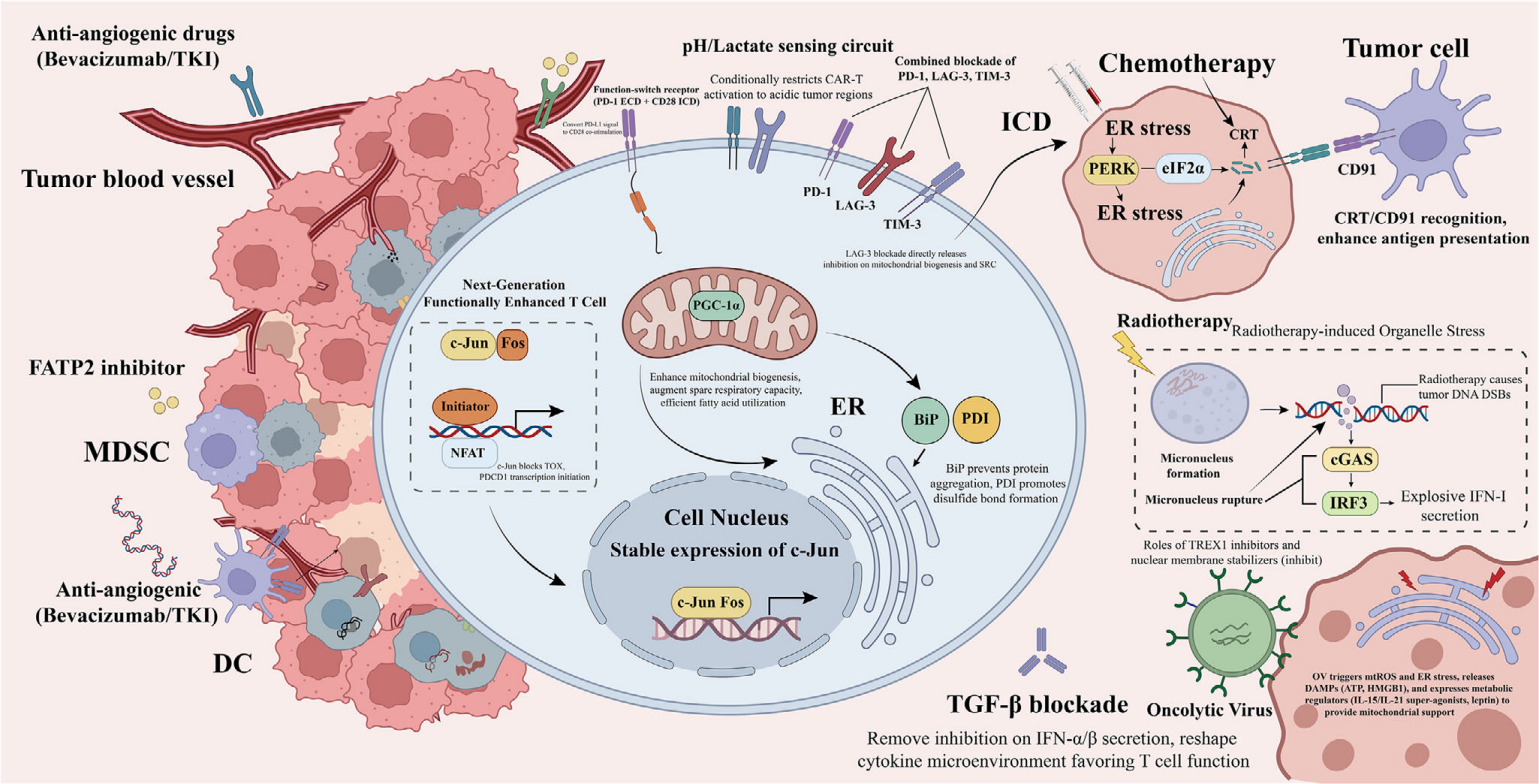
Intrinsic cellular engineering and multimodal combination therapeutic strategies driving comprehensive T cell resuscitation.

Beyond the well‐known *PD‐1*, next‐generation immune checkpoint molecules such as *LAG‐3* and *TIM‐3* can also suppress T‐cell responses by interfering with specific organelle functions. Research by Previte et al. confirmed that *LAG‐3* can directly limit mitochondrial biogenesis in naive T cells, maintaining them in a state of metabolic quiescence and impairing the SRC required for effector functions [261]. Further studies by Kim et al. elucidated that *LAG‐3* blocks the synergistic coupling between glycolysis and mitochondrial function in T cells by inhibiting *c‐Myc*‐dependent metabolic reprogramming. This finding suggests that *LAG‐3* not only impacts mitochondrial biogenesis but also deepens T‐cell functional suppression at the level of metabolic integration. Consequently, combined blockade of *PD‐1* and *LAG‐3/TIM‐3* can rescind key metabolic constraints on T cells from multiple dimensions, both restoring mitochondrial energy production and improving lysosome‐mediated metabolite turnover and signal degradation. This synergistic strategy facilitates the maximal restoration of metabolic fitness and functional plasticity in exhausted T cells, thereby overcoming the resistance commonly associated with monotherapies [262] (Figure 9).

## 11 Challenges and Future Directions

T cell exhaustion in the TME is a multi‐scale biological process in which suborganellar dysfunction serves as a key promoting link, presenting challenges for immunotherapy that go beyond traditional signal blockade. Although therapies targeting checkpoints such as PD‐1 can transiently restore partial T‐cell function, their efficacy remains extremely limited for terminally exhausted T cells that have undergone mtDNA mutations, respiratory chain supercomplex disassembly, or permanent loss of nuclear envelope stability. A core challenge is the lack of clinical tools capable of non‐invasive and dynamic assessment of T‐cell states. Current reliance on static indicators such as PD‐L1 expression or tumor mutational burden (TMB) fails to reflect critical functional states—such as mitochondrial membrane potential (Δ*ψ_m_*) or ER stress levels—within TILs, leading to an inability to identify exhaustion phenotypes at the patient level.

To bridge this translational gap, future clinical paradigms must prioritize the development of non‐invasive surrogate biomarkers that reflect subcellular T‐cell fitness. First, circulating metabolic signatures can serve as systemic indicators; for instance, an elevated kynurenine‐to‐tryptophan ratio has been clinically correlated with T‐cell metabolic exhaustion and poor prognosis in solid tumors [263]. Second, liquid biopsies focusing on T‐cell‐derived exosomes offer a window into intracellular stress. Exosomes enriched with fragmented mtDNA provide real‐time molecular readouts of mitochondrial organellar damage and tumor progression within the TME [264, 265]. Furthermore, advances in molecular imaging enable the spatial mapping of T‐cell states. Novel PET radiotracers, such as [18F]F‐AraG (which accumulates due to increased mitochondrial mass and mtDNA synthesis in activated T cells) or tracers targeting Granzyme B secretion, allow clinicians to non‐invasively assess TIL functionality and predict ICB responsiveness at extremely early stages [266, 267]. Mechanistically, super‐resolution live‐cell imaging and single‐cell metabolomics should be employed to elucidate the spatiotemporal sequence of signals driving pathologies such as mitochondrial fragmentation and ER distension across diverse tumor types. At the translational level, two engineering strategies warrant parallel exploration and optimization: first, the development of smart nano‐delivery systems for organelle‐specific targeting of antioxidants or metabolic modulators to reverse early dysfunction; and second, the utilization of gene editing or synthetic biological circuits to endow adoptive T cells with intrinsic metabolic fitness and stress resistance, thereby delaying the exhaustion process.

Consequently, future therapeutic paradigms necessitate a fundamental shift from attempting to reverse terminal exhaustion states toward intervening during early stages and correcting established dysfunctions. For instance, in desmoplastic tumors, matrix‐degrading agents could be combined with strategies to protect nuclear integrity; in tumors characterized by high metabolic competition, the relief of localized immunosuppression should be integrated with schemes to enhance intrinsic T‐cell bioenergetics. By intervening before functional impairment accumulates into irreversible structural damage and directly targeting the restoration of core organellar functional units, it will be possible to ultimately overcome the phenomenon of non‐responsiveness to immunotherapy.

## Funding

This work was funded by the Shanghai Health System Key Discipline Construction Project (Grant No.: 2024ZDXK0026), National Administration of Traditional Chinese Medicine, Comprehensive Office of Integrative Medicine “Flagship” Department Construction Project, 2024, Zhang Xiulan Charity Fund, Shandong Rural Revitalization Foundation, and Ren Nianrong Charity Fund, Shandong Rural Revitalization Foundation.

## Conflicts of Interest

The authors declare no conflict of interest.

## Data Availability

Data sharing not applicable to this article as no datasets were generated or analysed during the current study.
